# Spatial organization of the kelp microbiome at micron scales

**DOI:** 10.1186/s40168-022-01235-w

**Published:** 2022-03-24

**Authors:** S. Tabita Ramírez-Puebla, Brooke L. Weigel, Loretha Jack, Cathleen Schlundt, Catherine A. Pfister, Jessica L. Mark Welch

**Affiliations:** 1 Josephine Bay Paul Center for Comparative Molecular Biology and Evolution, Marine Biological Laboratory, Woods Hole, MA USA; 2 Present Address: The Forsyth Institute, Cambridge, MA USA; 3 Committee on Evolutionary Biology, University of Chicago, Chicago, IL USA; 4 Present Address: Friday Harbor Laboratories, University of Washington, Friday Harbor, WA USA; 5 Present Address: Wisconsin’s Green Fire, Rhinelander, WI USA; 6 Present Address: GEOMAR Helmholtz-Zentrum für Ozeanforschung Kiel, Kiel, Germany; 7 Department of Ecology and Evolution, University of Chicago, Chicago, IL USA

**Keywords:** Biogeography, Spatial structure, Polymicrobial interaction, CLASI-FISH, Host-microbe, Epiphytic, Endophytic, *Nereocystis luetkeana*

## Abstract

**Background:**

Elucidating the spatial structure of host-associated microbial communities is essential for understanding taxon-taxon interactions within the microbiota and between microbiota and host. Macroalgae are colonized by complex microbial communities, suggesting intimate symbioses that likely play key roles in both macroalgal and bacterial biology, yet little is known about the spatial organization of microbes associated with macroalgae. Canopy-forming kelp are ecologically significant, fixing teragrams of carbon per year in coastal kelp forest ecosystems. We characterized the micron-scale spatial organization of bacterial communities on blades of the kelp *Nereocystis luetkeana* using fluorescence in situ hybridization and spectral imaging with a probe set combining phylum-, class-, and genus-level probes to localize and identify > 90% of the microbial community.

**Results:**

We show that kelp blades host a dense microbial biofilm composed of disparate microbial taxa in close contact with one another. The biofilm is spatially differentiated, with clustered cells of the dominant symbiont *Granulosicoccus* sp. (*Gammaproteobacteria*) close to the kelp surface and filamentous *Bacteroidetes* and *Alphaproteobacteria* relatively more abundant near the biofilm-seawater interface. A community rich in *Bacteroidetes* colonized the interior of kelp tissues. Microbial cell density increased markedly along the length of the kelp blade, from sparse microbial colonization of newly produced tissues at the meristematic base of the blade to an abundant microbial biofilm on older tissues at the blade tip. Kelp from a declining population hosted fewer microbial cells compared to kelp from a stable population.

**Conclusions:**

Imaging revealed close association, at micrometer scales, of different microbial taxa with one another and with the host. This spatial organization creates the conditions necessary for metabolic exchange among microbes and between host and microbiota, such as provisioning of organic carbon to the microbiota and impacts of microbial nitrogen metabolisms on host kelp. The biofilm coating the surface of the kelp blade is well-positioned to mediate interactions between the host and surrounding organisms and to modulate the chemistry of the surrounding water column. The high density of microbial cells on kelp blades (10^5^–10^7^ cells/cm^2^), combined with the immense surface area of kelp forests, indicates that biogeochemical functions of the kelp microbiome may play an important role in coastal ecosystems.

Video abstract

**Supplementary Information:**

The online version contains supplementary material available at 10.1186/s40168-022-01235-w.

## Background

Seaweeds are a diverse group of algae that evolved multicellularity more than a billion years ago in an ocean populated by diverse microbial lineages [1]. Just as animals and plants, evolving in a microbial world, developed interdependent relationships with an array of bacteria [2, 3], so bacteria associated with seaweed surfaces play critical roles in host biology, for example by synthesizing essential vitamins, preventing biofouling, and providing signals essential for normal host development [4]. Bacterial-macroalgal symbioses are major contributors to coastal nutrient cycling, as bacteria consume and remineralize organic carbon secreted by the host [5–8] and may provision resources like nitrogen and vitamins to their host [1, 4, 9]. Thus, the bacterial-macroalgal symbiosis is an ecologically important and potentially tractable model system for investigating the principles of host-microbe biology.

While the cast of characters in the macroalgal-bacterial symbiosis is becoming clear, less clear is how the members of the community interact with one another and how intimately they associate with their host. The bacterial colonizers that thrive on macroalgae are a subset of those found in seawater as a whole and have been shown to be both host-specific and also variable over time and space [10–13]. They include members of such disparate bacterial groups as *Planctomycetes*, *Verrucomicrobia*, *Bacteroidetes*, and *Alpha*- and *Gamma*-proteobacteria. Unresolved questions include whether each of these taxa interacts directly with the host surface, are present endophytically, or are epibionts on one another; whether surface biofilms are characterized by large single-taxon patches or by cell-to-cell mixing between taxa; how microbial cell density changes with host tissue age; and whether the biofilm structure changes in declining vs. healthy macroalgal populations.

Addressing these questions will require new kinds of information that are spatially resolved at the scale at which microbes interact with one another. Microbes interact by secretion of metabolites, inhibitors, and signaling molecules as well as by direct contact. In open systems with fluid flow, these interactions occur primarily between cells located no more than a few micrometers to tens of micrometers apart [14–17]. Thus, the positioning of bacteria at micrometer scales reveals which partners are potentially available for interaction. Sequencing approaches have been enormously fruitful in advancing our understanding of microbial communities but have the disadvantage that the sample must be homogenized for extraction of DNA, RNA, and other molecules of interest, thereby destroying the information contained in the micron-scale spatial structure of the sample. Imaging approaches, by contrast, preserve this information and permit us to ask an expanded range of questions about host-microbe and microbe-microbe interactions.

In this study we investigated the micron-scale spatial organization of microbial communities living on photosynthetic blades of bull kelp, *Nereocystis luetkeana* (order *Laminariales*), using combinatorial labeling and spectral imaging–fluorescence in situ hybridization (CLASI-FISH [18, 19]). This annual kelp displays extraordinarily high growth rates during the spring and summer, with photosynthetic kelp blades growing outwards from the kelp thallus at rates of 0.5–2 cm per day [10]. We leveraged this rapid growth to assess microbial spatial organization at two different successional stages in a single sampling: initial colonizers on tissues only a few days old at the base of the blade, compared to later stages of growth and development of the biofilm on tissues weeks to months old, at the tip [10]. In addition, we assessed the relationship between the health of the kelp and the structure of its microbiome by comparing biofilm structure on a healthy *N. luetkeana* population to a geographically distinct population that has been in decline in recent years [20, 21]. We show that pioneer taxa in the kelp microbiome provide surfaces for later colonization of a more complex microbiota, that taxon distribution is mixed at micron scales and thus provides the opportunity for syntrophy, and that the microbiota in a declining population has both low density and low complexity.

## Results

### Relative abundance of kelp-associated microbial community and CLASI-FISH strategy

**Fig. 1:**
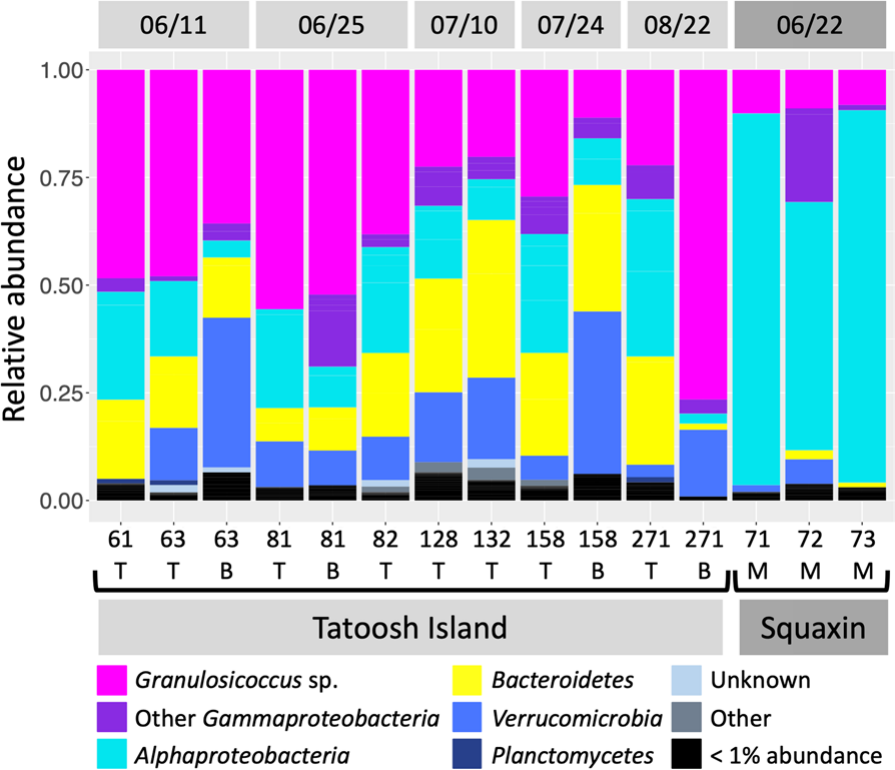
Relative abundance of bacterial taxa on the kelp *N. luetkeana*. Sequences are grouped by phylum, class and genus-level taxonomy to show taxa detected by our CLASI-FISH probe set. 16S rRNA gene sequencing showed that bacterial composition was broadly consistent throughout the summer. The most abundant taxa were *Gammaproteobacteria*, *Alphaproteobacteria*, *Bacteroidetes*, and *Verrucomicrobia*. The gammaproteobacterial genus *Granulosicoccus* was highly abundant in samples from Tatoosh Island, while *Alphaproteobacteria* were dominant at Squaxin Island. Collection date is shown at the top, and collection site is shown at the bottom. Sample numbers and portion of the kelp blade sampled (T = tip; B = base; M = middle) are indicated in the *x*-axis labels

To investigate the community structure of the kelp microbiota over time and in kelp populations at different locations in Washington State, we collected kelp blade tissue samples from two sites reported in Weigel and Pfister [10]: Squaxin Island, the site of a declining kelp population in Southern Puget Sound [21], and Tatoosh Island, the site of a persistent kelp population on the outer coast of the Olympic Peninsula. To assess the microbial spatial organization at two different successional stages, we collected 2 samples per individual at Tatoosh Island: tissue only a few days old at the meristematic base of the blade, and tissue weeks to months old, at the tip. Characterizing the microbiome of *N. luetkeana* with 16S rRNA gene sequencing, as previously reported [10], revealed a community that was diverse but composed of the same major taxa at all sampled time points and both locations (Fig. 1). The most abundant genus-level taxon was *Granulosicoccus* sp. (*Gammaproteobacteria*), which accounted for an average of 38.3% (± 18.6%, std. error) of the microbial community in samples from Tatoosh Island (Fig. 1); other major taxa included *Alphaproteobacteria* (17.9 ± 10.6 %), *Bacteroidetes* (19.6 ± 0.1%), *Verrucomicrobia* (14.5 ± 0.12%), and *Planctomycetes* (0.90 ± 0.01%). In contrast, samples from the declining kelp population at Squaxin Island were dominated by the alphaproteobacterium *Robiginitomaculum* sp. (76.7 ± 16.5%), while *Granulosicoccus* sp. (9.1 ± 0.98%), *Bacteroidetes* (1.7 ± 0.91%), and *Verrucomicrobia* (2.7 ± 2.6%) were much less abundant than at Tatoosh Island (Fig. 1).

We designed a probe set for CLASI-FISH to visualize all bacterial types in the sample simultaneously. The specificity of the probes ranged from domain to phylum, class, and genus, so that most cells were identified by hybridization of two or three probes at different phylogenetic levels of specificity (Table 1). This phylogenetically nested probing strategy permitted imaging that was both comprehensive, visualizing nearly all bacterial cells, and specific, differentiating the major bacterial groups including *Alphaproteobacteria*, *Gammaproteobacteria*, *Bacteroidetes*, *Verrucomicrobia*, and genus *Granulosicoccu*s within the *Gammaproteobacteria* (see Additional file 1: Fig. S1). We carried out CLASI-FISH and imaging on a total of 15 samples: 3 from Squaxin Island, collected on 21 June 2017, and 12 from Tatoosh Island, collected at five time points spaced roughly 2 weeks apart from 11 June–22 August 2017 (see Additional file 2: Table S1). Imaging of the kelp-associated microbial community presented issues of sample preparation. We used several complementary sample preparation and imaging approaches that allowed us to minimize distortion and preserve spatial organization (see Additional file 3: Fig. S2).

**Table 1:** FISH probes used in this study

Probe	Target taxon	Probe sequence 5′–3′	Fluorophore	Reference
Eub338-I	*Bacteria*	GCTGCCTCCCGTAGGAGT	Dy490 or Atto532	[22]
Eub338-II	*Planctomycetes*	GCAGCCACCCGTAGGTGT	Dy415	[23]
Eub338-III	*Verrucomicrobia*	GCTGCCACCCGTAGGTGT	Dy415	[23]
Alf968	*Alphaproteobacteria*	GGTAAGGTTCTGCGCGTT	Dy490 or Atto620	[24]
Gam42a	*Gammaproteobacteria*	GCCTTCCCACATCGTTT	Cy5	[25]
Bac1058	*Bacteroidetes*	TGAATGGCTGCTTCCAAGCCAACA	Rhodamine Red-X	[26]
Gran737	*Granulosicoccus* sp.	TCAGCGTCAGTATTGTTCCAGA	Texas Red-X	This study
Gran670	*Granulosicoccus* sp.	CACCGCTACACCCGGAATTCCGC	Texas Red-X	This study

Probe name, target taxon, 5′–3′ sequence, fluorophores used, and references are indicated

### Epiphytic and endophytic microbial communities associated with kelp blades

**Fig. 2:**
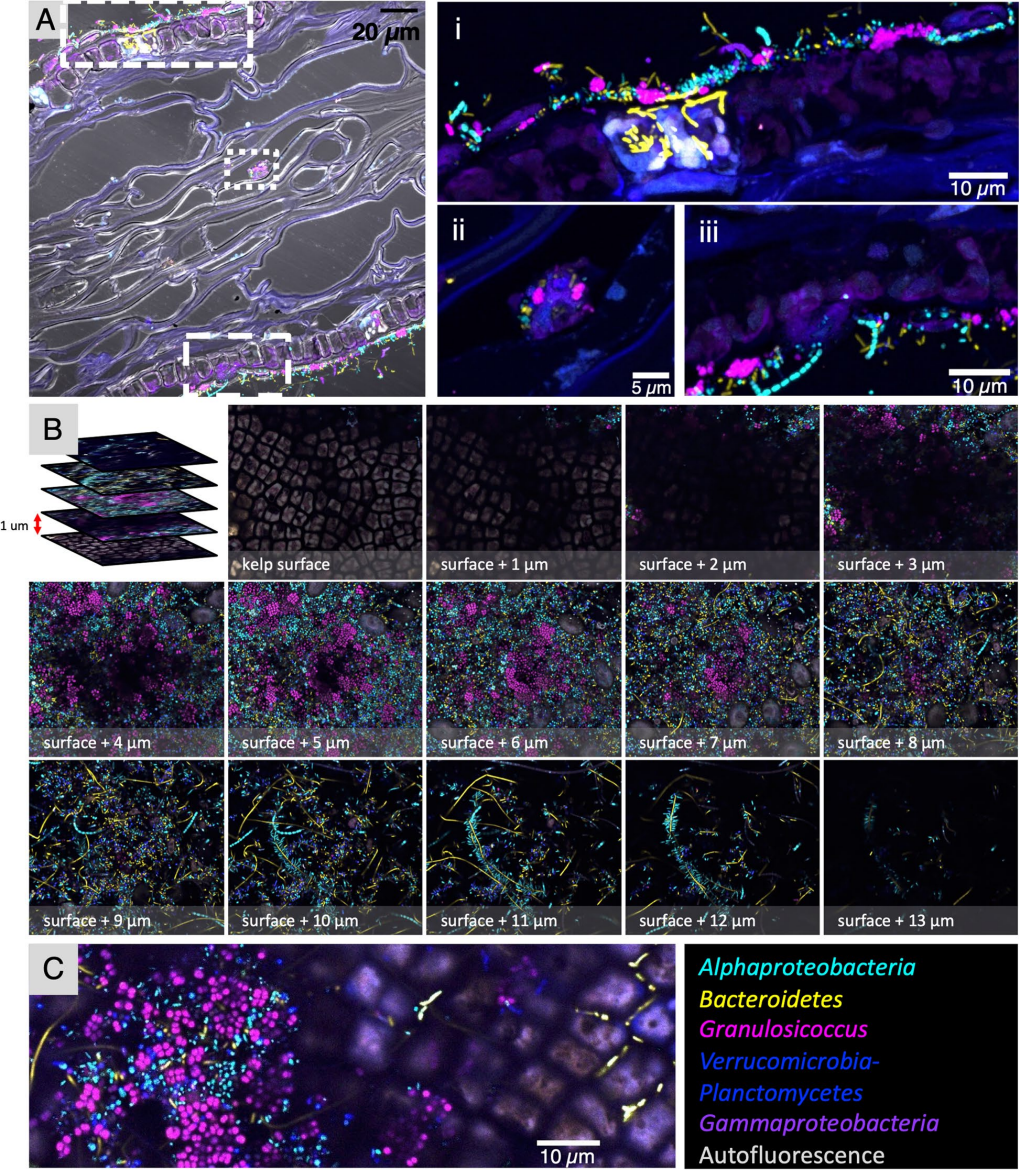
Cross-sectional, whole-mount, and oblique optical section images give different views of the biofilm on kelp*. N. luetkeana* blades were subjected to CLASI-FISH with a probe set for the 5 major bacterial groups. **A** Cross-sectional image of a kelp blade embedded in methacrylate. Merged transmitted light and confocal images show the microbial biofilm on both sides of the kelp blade, with some microbial cells in the center. (i), (ii), and (iii) are enlarged images of the dashed rectangles in panel (**A**). *Bacteroidetes* rods within the autofluorescent region of kelp tissue in panel (i) fluoresce more brightly than rods in the surface biofilm and therefore appear overexposed in the image. **B** Whole-mount preparation imaged as a *z*-stack; planes 1 micrometer apart in the z dimension are shown. **C** Oblique optical section showing the microbial biofilm at left and kelp surface at right. *Bacteroidetes* rods are visible between kelp surface cells (right)

Imaging of cross sections of kelp embedded in methacrylate enabled us to visualize the relationship of the microbiota to the underlying kelp tissue (Fig. 2A). The kelp tissue was visible in a transmitted-light image as a series of large irregularly shaped chambers (sieve tubes), with a row of oblong photosynthetic cells along both the upper and the lower surface of the blade (Fig. 2A). The microbes were located primarily in a dense layer several micrometers thick on the exterior of both the upper and lower surface. Imaging at a magnification sufficient to visualize individual bacterial cells showed each taxon as single cells or small clumps, often immediately adjacent to cells of different taxonomic identification (Fig. 2A, insets i–iii). In addition to the surface layer, some bacteria were present in the interior of the blade; a subset of the community consisting largely of *Bacteroidetes* rods can be seen within the kelp tissue immediately beneath the surface layer (Fig. 2Ai), and a small cluster of mixed composition is visible near the center of the image (Fig. 2Aii). Thus, a variety of interactions between microbiota and host were visualized in a cross-sectional view.

By contrast, placing a whole mount of a kelp blade flat on a microscope slide permitted imaging of a vertical series (or *z*-stack) of images from the surface of the kelp blade through the biofilm (Fig. 2B). This whole-mount imaging revealed the relationship of microbial cells to one another and the changing composition of the community as a function of distance from the kelp blade. Moving up from the plane in which the kelp photosynthetic cells are visible (Fig. 2B), serial optical sections show first a largely fluorescence-free region a few micrometers thick and then dense colonization by a mixed microbial community. At approximately 3 to 7 μm from the surface of the blade, clusters of *Granulosicoccus* cells are prominent and diatoms are visible (Fig. 2B). In the region approximately 8 to 12 μm from the blade surface, filaments of *Bacteroidetes* and chains of *Alphaproteobacteria* are a more prominent part of the community. Non-filamentous cells of *Alphaproteobacteria*, *Bacteroidetes*, and *Verrucomicrobia* or *Planctomycetes* are present throughout the biofilm. The whole-mount images, like the cross sections, show that cells of disparate taxa are directly adjacent to one another in the kelp surface biofilm.

In a perfectly flat whole-mount image one sees either the algal surface or the microbiota but not both, unless the microbiota has invaded into the tissue. In many whole-mount images, however, the sample is tilted relative to the plane of imaging (Additional file 3: Fig. S2B), such that the confocal microscope image is an optical section through the sample at an oblique angle and a single plane of focus captures both the kelp blade and the overlying microbial community. In the example in Fig. 2C, kelp cells are visible as large (~ 5 μm × 10 μm) oblongs at the right; *Bacteroidetes* spp. are intercalated between the kelp cells. In the center of the image scattered taxonomically mixed bacteria are located on or between the kelp cells, while at the left-hand side of the image the full microbial community is visible. As most whole-mount preparations are not entirely flat, many images represent to one degree or another an oblique-angle view of the material.

### Bacteria on kelp blades form mixed epiphytic communities whose abundance is dependent on the age of the underlying tissue

**Fig. 3:**
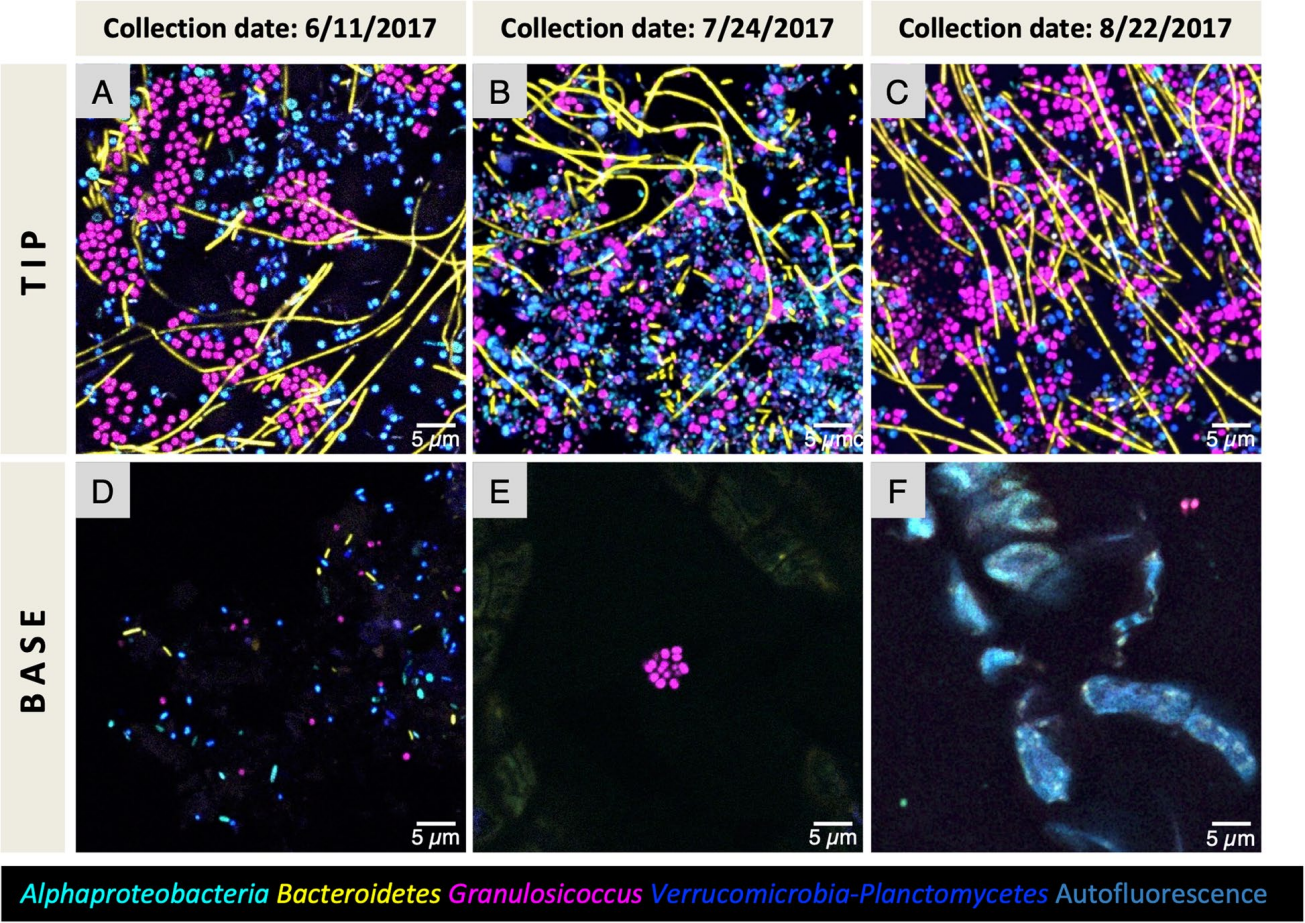
Bacterial cell abundance of the surface biofilms of old and young tissue. Whole-mount images of kelp blade tip tissues (old) and base tissues (young) collected from the same kelp frond. Three individuals from different collection dates are shown. Older tissue from the tip of the kelp blade (**A**, **B**, **C**) is densely colonized compared to young tissue from the base of the blade (**D**, **E**, **F**). The same pattern is observed throughout the summer

Due to the high growth rate of the kelp thallus, producing multiple centimeters of new tissue in a single day [10], it is possible to study how spatial structure and diversity of the microbial community differ between newly produced tissue vs. tissue that is weeks to months old. Although the microbial community on older tissue contains more diversity at the amplicon sequence variant level [10], the composition and relative abundances of major taxa are broadly consistent across young and old tissues (Fig. 1). However, imaging showed a higher density of colonization at the tip of the blade, which is months old, compared to the tissue near the basal meristem, where newly produced tissue is only days old (Fig. 3; Additional file 4: Fig. S3A). At the tip (Fig. 3A–C), microbial cells form a dense biofilm, whereas at the base only scattered microbial cells are visible (Fig. 3D–F). This pattern is observed in samples collected throughout the summer (Fig. 3). Cell counts of each bacterial type are shown in Table 2; the mean density of bacterial cells observed in images from the base was 1.12 × 10^5^ and in images from the tip was 2.17 × 10^7^ cells/cm^2^.

**Table 2:** Cell size and density of major taxa

Taxon	Cell area μm^2^ (SD)	Tatoosh tip (old) cell count (SD)	Tatoosh tip (old) cells/cm^2^	Tatoosh base (young) cell count (SD)	Tatoosh base (young) cells/cm^2^	Squaxin cell count (SD)	Squaxin cells/cm^2^	Ratio tip: base	Ratio tip: Squaxin
*Granulosicoccus*	0.60 (0.16)	2921.2 (1853.5)	6.47 × 10^6^	24.9 (29.9)	5.50 × 10^4^	15.3 (17.5)	3.38 × 10^4^	118	192
*Verrucomicrobia + Planctomycetes*	0.53 (0.08)	2449.9 (1174.0)	5.42 × 10^6^	5.4 (9.2)	1.18 × 10^4^	13.4 (16.0)	2.96 × 10^4^	458	183
*Alphaproteobacteria*	0.30 (0.10)	2879.0 (1783.1)	6.37 × 10^6^	10.0 (20.4)	2.21 × 10^4^	54.7 (85.7)	1.21 × 10^5^	288	53
*Bacteroidetes*	1.62 (0.50)	1645.4 (982.6)	3.64 × 10^6^	10.5 (21.3)	2.31 × 10^4^	6.3 (13.3)	1.38 × 10^4^	157	263
Four major taxa	NA	9813.2 (4058.7)	2.17 × 10^7^	50.7 (49.1)	1.12 × 10^5^	89.6 (121.0)	1.98 × 10^5^	194	110

Analysis was performed across 10 individuals (2–11 images each; *n* = 56 images); each image measured 212.55 × 212.55 μm. Mean cell area, mean cell number (count) per image, and calculated cell densities are given for each bacterial taxon for samples from the tip (older tissue) and base (young tissue) of blades at Tatoosh Island and from the center of blades at Squaxin Island. Standard deviation is shown in parentheses

The spatial arrangement of bacterial cells provides information about the process of colonization. The major types of cells—*Granulosicoccus*, *Alphaproteobacteria*, *Bacteroidetes*, and *Verrucomicrobia/Planctomycetes*—were all observed adhered to young kelp tissue as single cells independent of one another (Fig. 3D–F). This arrangement suggests that each of these types is capable of independent binding to the kelp surface. Images of the more complex and well-developed biofilm at the tip, however, show bacteria adhered to other bacteria rather than to the kelp itself. Alphaproteobacterial rods, for example, were observed surrounding a *Bacteroidetes* filament and adhering to it at their tips, in a test tube brush formation (Fig. 2B). Thus, our images provide evidence that the pioneer colonizers of the kelp surface can colonize directly and independently but that later colonizers may bind to the early colonizers rather than directly to the kelp surface.

**Fig. 4:**
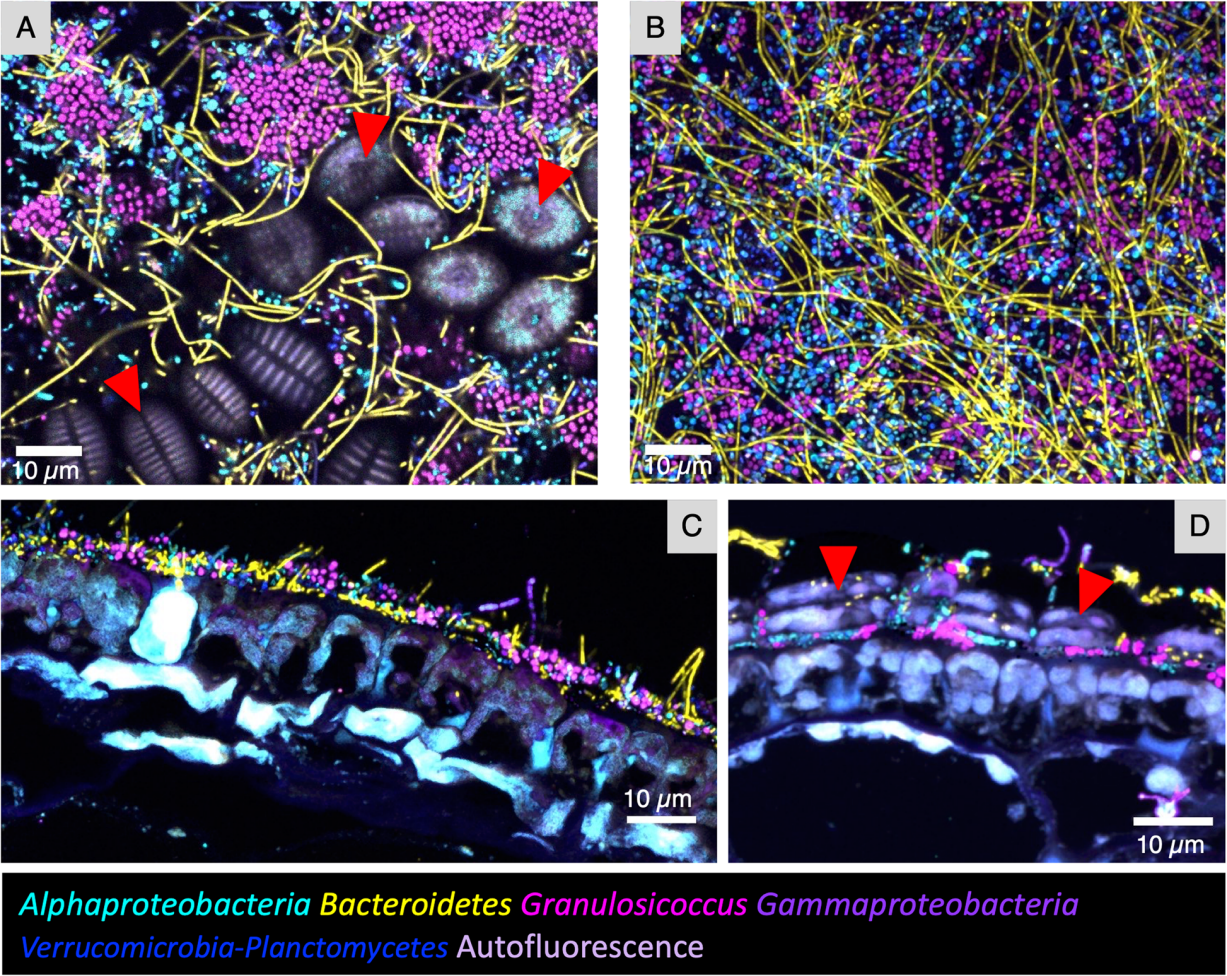
Spatial structure of the epiphytic microbial community at the tip of *N. luetkeana* blades. Bacteria at the tip of kelp blades form a dense biofilm. **A** and **B** show whole-mount images of samples collected on different dates; **C** is a cross section showing the thickness of the surface biofilm relative to the kelp cell surface. Microorganisms are intermixed, always within 10 microns of other taxa, and often directly adjacent to cells of disparate taxa or diatoms (red arrowheads). *Granulosicoccus* aggregate in clusters while other taxa are more dispersed. Abundant *Bacteroidetes* filaments appear to be lying across the other taxa in (**A**) and (**B**). In the cross section (**C**), filaments of *Bacteroidetes* and *Gammaproteobacteria* project into the water column. **D** Cross section showing diatoms embedded within the bacterial biofilm

**Fig. 5:**
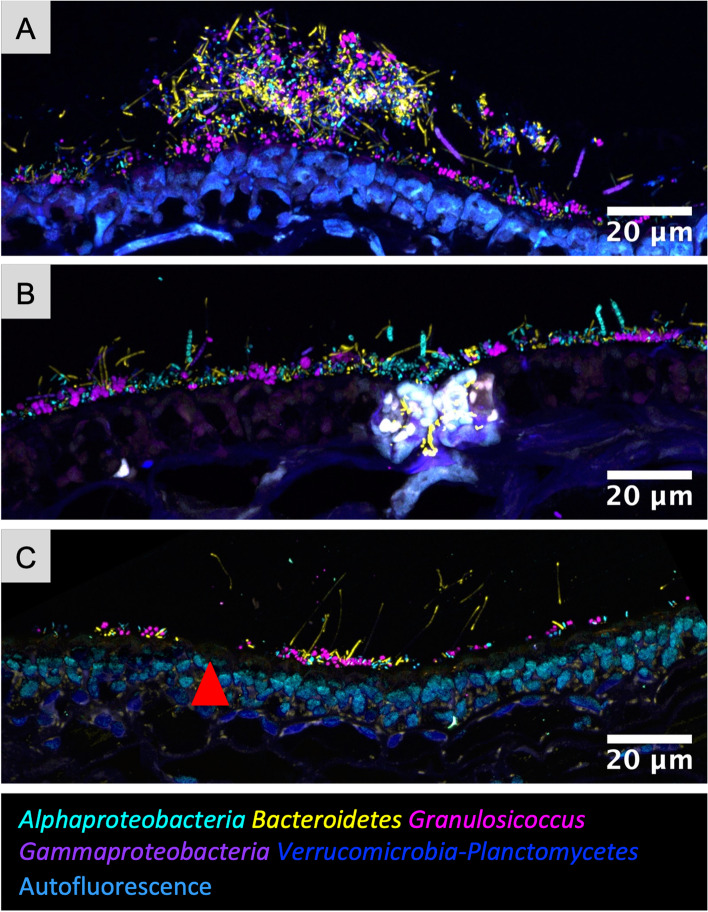
Variation of biofilm thickness. Cross-sectional images showing the variation of the biofilm thickness within and among samples. **A** A patch of biofilm up to 32μm thick with adjacent thinner biofilm of 3 to 6 μm. **B** The most common thickness observed in the kelp biofilm was 3 to 7 μm, with chains and filaments extending further into the water column. **C** A sparse biofilm including a region with no visible biofilm (red arrowhead)

Typical images of the tip community (Fig. 4) show that bacteria at the tip of kelp blades are mixed at micron scales. *Granulosicoccus*, the most abundant genus revealed by 16S rRNA gene sequencing, formed patches or clusters up to 15 μm in diameter in some samples (Fig. 4A); close inspection reveals cells of other taxa nestled within the clusters (Fig. 4A). The frequent appearance of *Granulosicoccus* as pairs or tetrads of cells (Figs. 2C and 3A, C, E, and F) suggests cell division and growth of the *Granulosicoccus* as a mechanism for the formation of clusters, although accretion or aggregation of existing cells is another possible mechanism. *Alphaproteobacteria*, *Bacteroidetes* rods, *Verrucomicrobia*, and *Planctomycetes* did not form large clusters but instead are intermixed in the biofilm. In whole-mount preparations, *Bacteroidetes* filaments lie on top of the other bacteria (Figs. 3A–C and 4A, B), suggesting that they are in a different level of the biofilm and become flattened onto the sample during the FISH and mounting procedure. This observation is reinforced by cross-sectional images, which show a biofilm typically 3 to 7 μm thick, with filaments projecting approximately 10 μm from the biofilm before either ending or moving out of the plane of the section (Figs. 4C and 5). The considerable length of the *Bacteroidetes* filaments viewed in whole-mount preparations (e.g., 50 μm or more in Fig. 3) again suggests the growth of this taxon in situ.

In addition to a dense bacterial biofilm, colonies of diatoms were observed in samples collected on Tatoosh Island in June and July (Fig. 4A). In a cross-sectional image, diatoms can be seen embedded within the biofilm and a layer of bacteria is observed between them and the kelp tissue (Fig. 4D), suggesting that bacteria colonized the host before adhesion of the diatoms. No specific association between diatoms and particular bacterial taxa was observed.

The thickness of the bacterial biofilm is best observed in cross-sectional images, which are not subject to the flattening that occurs in whole mounts, and was variable both between and within sections (Fig. 5). The most frequently observed thickness ranged between 3 and 7 μm while the thickest patch measured was 32 μm in one sample (Fig. 5A). Regions of 0-μm thickness were also observed (Fig. 5C). We did not detect any trend toward either increasing or decreasing thickness of the biofilm over the growing season. This relatively consistent thickness suggests that the addition of bacteria by colonization and replication is balanced by lateral expansion of the blade surface or by a process of biofilm loss, perhaps due to shedding of material from the surface of the kelp tissue.

### Endophytic bacteria and direct interactions with the blade surface

**Fig. 6:**
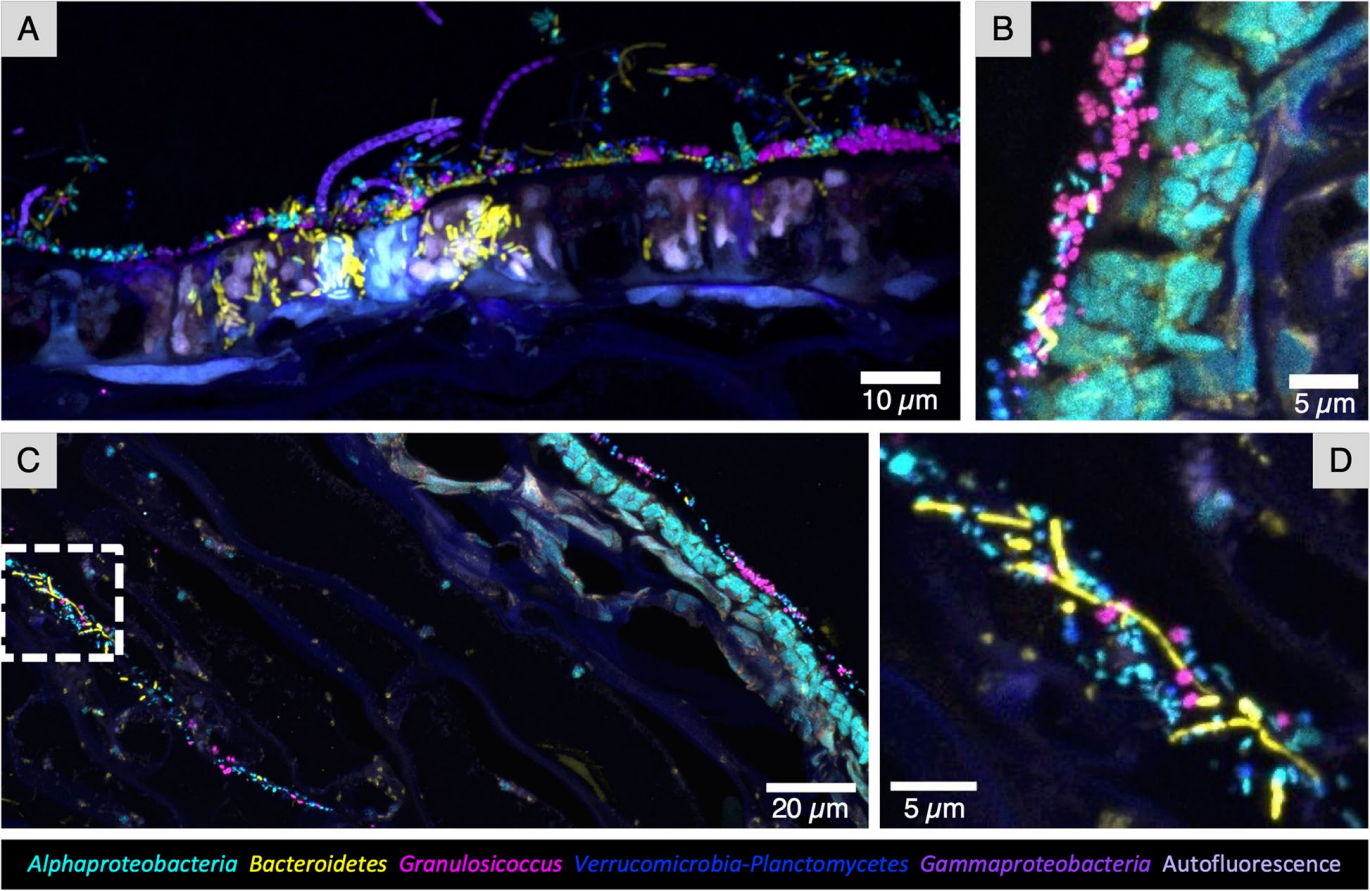
Endophytic bacteria of *N. luetkeana*. **A** Cross section showing *Bacteroidetes* rods colonizing intercellular spaces of brightly autofluorescent kelp surface cells. **B** A region in which the biofilm is directly adjacent to the kelp tissue and some *Granulosicoccus* are observed between kelp cells. **C** Bacteria were also detected colonizing deeper areas of the tissue, in this instance around 120 μm from the surface. **D** Enlarged image of the dashed rectangle in (**C**)

Imaging of cross sections permitted the identification of bacteria inside kelp tissue. *Bacteroidetes* rods and, less abundantly, other members of the community were observed colonizing intercellular spaces of the outermost layer of kelp cells in samples from July (Figs. 2A, C and 6). These endophytic *Bacteroidetes* were located adjacent to kelp cells that showed strong autofluorescence, suggesting a different physiological state from the rest of the thallus: either higher metabolic activity or a process of tissue damage and repair. Microbes occasionally colonized the surface layer of cells directly (Fig. 6B), but in contrast to the *Bacteroidetes*-rich invasion into highly autofluorescent regions, no obvious preference for any underlying morphology of the kelp was detected. We also observed a mixed microbial community forming small clusters on the interior of the frond (Fig. 2Aii) or forming a strand running through the kelp inner tissue (Fig. 6C, D). While our probe detected *Bacteroidetes* at the phylum level, 16S sequencing results showed that *Saprospiraceae* and *Flavobacteriaceae* were the two most common families within this phylum in these samples [10]. Determining the identity and functional role of these apparent endophytic bacteria is an interesting avenue for future work.

### Unipolar labeling of adherent *Alphaproteobacteria* with wheat germ agglutinin

**Fig. 7:**
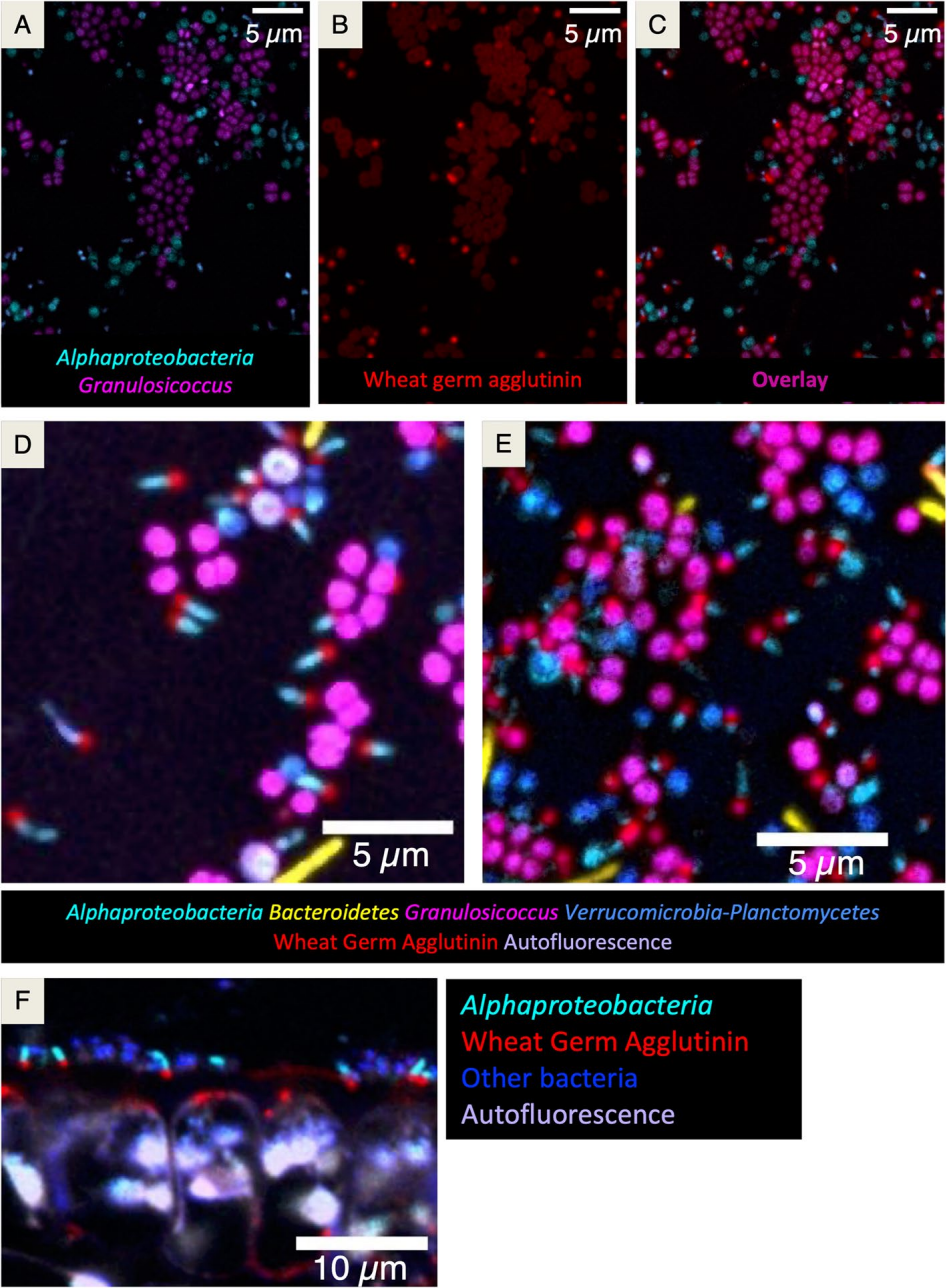
Unipolar labeling of adherent *Alphaproteobacteria* by wheat germ agglutinin. Wheat germ agglutinin was used to stain N-acetylglucosamine and N-acetylmuramic acid residues. Staining was observed on *Alphaproteobacteria* rods at only one end, showing apparent polarity with respect to the cells. **A** Hybridization of a kelp sample with probes for *Alphaproteobacteria* (cyan) and *Granulosicoccus* (magenta). **B** Signal from fluorophore-labeled WGA (red) in the same field of view as (**A**). **C** Merged image of A and B showing that WGA staining was observed in cells hybridizing with the *Granulosicoccus* and *Alphaproteobacteria* probes. WGA staining was detected surrounding the *Granulosicoccus* cells while the stained *Alphaproteobacteria* showed fluorescence at only one end of the cell. WGA signal associated with *Alphaproteobacteria* was brighter and more localized than that associated with *Granulosicoccus*. **D** and **E** Representative images of FISH on whole-mount samples from kelp blades collected in different months from Tatoosh Island. **F** Cross-sectional image showing *Alphaproteobacteria* rods with the polar polysaccharide end adjacent to the kelp surface

**Fig. 8:**
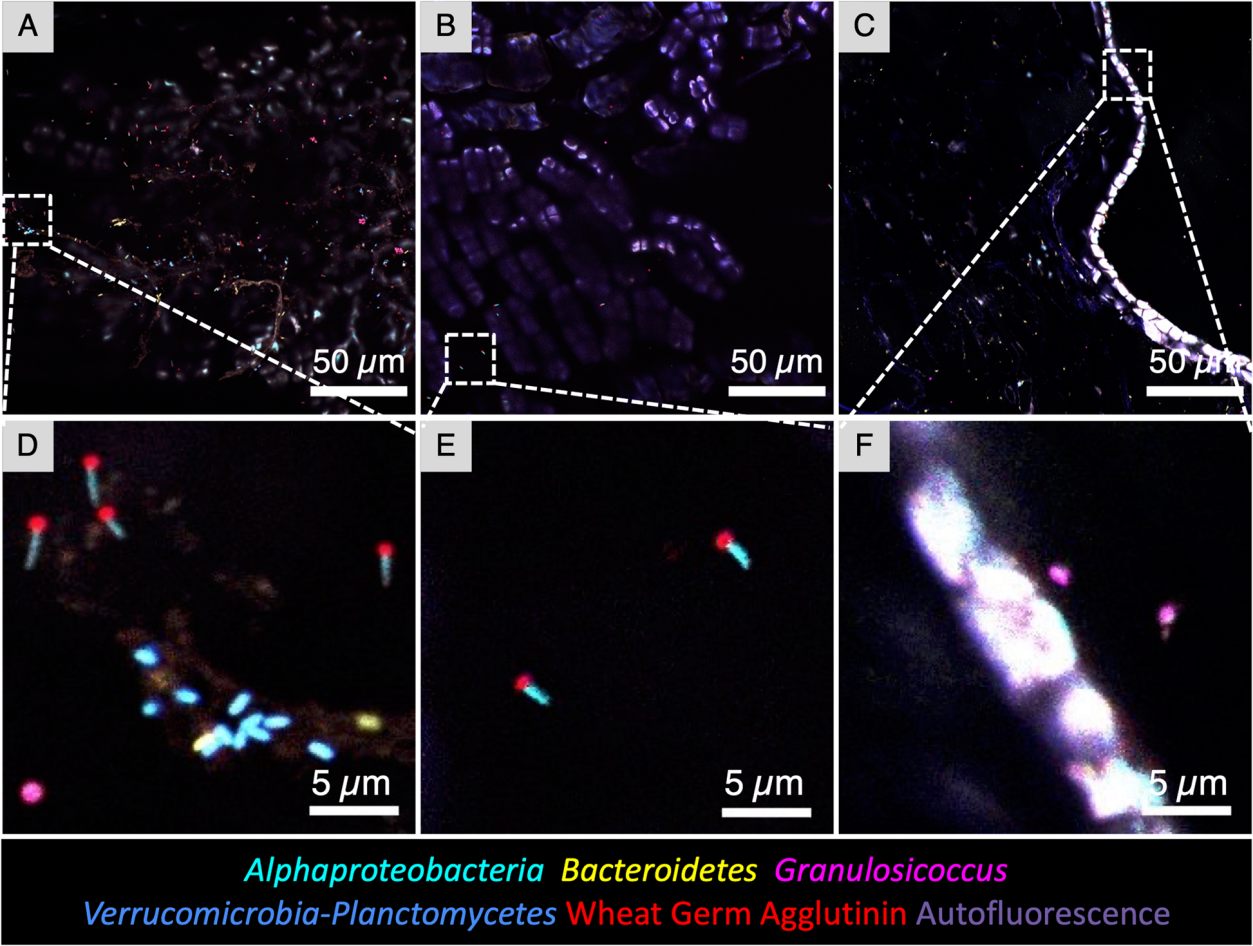
Low microbial density on kelp blades from a declining kelp population at Squaxin Island. **A** and **B** Whole mount FISH showing sparse bacteria on the mid-blade kelp surface. **C** Cross section in which no dense biofilm was observed on the surface, but a few bacteria were visible. Strong autofluorescence of kelp cells was observed. **D**, **E**, and **F** are enlarged images of the dashed squares in (**A**), (**B**), and (**C**), respectively

Wheat germ agglutinin (WGA) is a lectin that binds to N-acetylglucosamine and N-acetylmuramic acid residues that can be present both in bacterial cell walls and in host mucilage secretions. We stained samples with fluorophore-labeled WGA and observed staining in spots on the kelp tissue itself, in cells hybridizing with the *Granulosicoccus* probe, and most intriguingly, on cells hybridizing with the *Alphaproteobacteria* probe that reacted asymmetrically with the WGA, showing fluorescence at only one end of the cell (Fig. 7). This pattern was observed across multiple individual kelp collected in different months and sites (Fig. 7D, E) including on the kelp from Squaxin Island (Fig. 8D, E). In cross-sectional images, the WGA signals were present at the end of the cell that was in contact with the kelp surface (Fig. 7F) suggesting a potential role of the WGA-stained structure in the adhesion of the microbe to the kelp. These microbes may be members of the genus *Robiginitomaculum*, as one *Alphaproteobacteria* ASV that matched with 96% sequence identity to *Robiginitomaculum antarcticum* had 76% relative abundance at Squaxin Island. This genus has been shown to produce thin, stalk-like structures called prostheca at one end of the cell [27], which might be the component that is being labelled by the WGA probe. Further research may determine whether this structure is involved in attachment to the kelp surface.

### Bacterial density differences between samples from healthy and declining kelp populations

Imaging revealed dramatically lower densities of microbial cells on Squaxin Island kelp (Fig. 8) compared to the tip of Tatoosh Island kelp blades. The absolute abundance of bacterial cells was two orders of magnitude higher in samples collected from the persistent kelp forest located on the outer Pacific Ocean site of Tatoosh Island than in the locally declining population at Squaxin Island [21], located in Southern Puget Sound (Table 2). This differential abundance occurred for all taxa but was less extreme for *Alphaproteobacteria* (Table 2) because of their high relative abundance in kelp from the Squaxin site. Kelp at Squaxin Island were sampled from the middle of the blade, where the tissue was approximately 2 months old, similar to the age of the tissue collected from the tip of the kelp blade on Tatoosh Island (Additional file 2: Table S1). The density of microbes imaged at the base of Tatoosh Island blades resembled that at the middle of Squaxin blades (compare Figs. 3D–F and 8). Thus, samples from the base of the Tatoosh blades that were less than 1 week old had similar microbial densities to these 2-month-old biofilms from the declining kelp population on Squaxin Island, suggesting that the host-microbial symbiosis is actively maintained by the host and/or microbial partners and collapses in the absence of host health, or that environmental differences between these two distant sites exerted a significant influence on the density of the microbial biofilm. Further research is necessary to determine the cause of reduced microbial cell densities on kelp from Southern Puget Sound.

### Quantification and micron-scale spatial arrangement

Quantification of cell abundance in FISH images showed that mean abundances as assessed by imaging were broadly consistent with relative abundance estimates from sequencing, and that community composition in the biofilm closest to the kelp surface differed from that near the water column. We calculated the mean bacterial abundance on blades of 10 *Nereocystis* individuals. *Granulosicoccus* was the most abundant taxon both at the tip and at the base of Tatoosh samples and *Alphaproteobacteria* was the most abundant on Squaxin samples, consistent with the 16S rRNA gene sequencing (Table 2, Additional file 4: Fig. S3B). Cell densities of the major taxa varied according to the tissue age and collection site. The blade tip presented the highest density (2.17 × 10^7^ cells/cm^2^), which is 110 and 194 times higher than from the mid-blade at Squaxin (1.98 × 10^5^ cells/cm^2^) and Tatoosh blade base (1.12 × 10^5^ cells/cm^2^) densities, respectively. Mean bacterial abundance was higher in the layers of the biofilm closer to the kelp surface compared to the layers closer to the water column (Fig. 2B; Additional file 5: Fig. S4). Community composition shifted with distance from the host, with *Granulosicoccus* detected primarily close to the kelp surface. Mean cell area differed among taxa, with *Granulosicoccus* measuring 0.60 μm^2^, *Verrucomicrobia* 0.53 μm^2^, *Alphaproteobacteria* 0.30 μm^2^, and *Bacteroidetes* 1.62 μm^2^ (Table 2).

Quantitative analysis of micron-scale spatial arrangement showed auto-correlation at short distances, and weaker cross-correlation of taxa with respect to one another in these images. We measured auto- and cross-correlation using linear dipole analysis, a stereological method for determining pair cross-correlation functions over a range of distances, as implemented for image analysis using the program *daime* [28]. We analyzed within- and between-taxon associations for *Granulosicoccus*, *Alphaproteobacteria*, and the *Verrucomicrobia*-*Planctomycetes* (hereafter referred to as simply *Verrucomicrobia*) taxa for each of 8 kelp individuals using whole-mount images. Results showed that all three taxa were positively autocorrelated. *Granulosicoccus* had the highest autocorrelation (Table 3, Additional file 6: Fig. S5A) which reached a maximum at 1.5 μm and reached half the maximum amplitude at 1 to 3 μm, reflecting the size of clusters visible in the images. *Verrucomicrobia* and *Alphaproteobacteria* cells showed self-attraction at distances of 1 to 3 μm and 1 to 4.5 μm, respectively (Table 3, Additional file 6: Fig. S5). Values of cross-correlation between taxa were lower (Additional file 6: Fig. S5B). Cross-correlation between *Alphaproteobacteria* and *Verrucomicrobia* peaked at 1.5 μm. Only slightly elevated cross-correlation was observed between *Granulosicoccus* and *Verrucomicrobia* and between *Granulosicoccus* and *Alphaproteobacteria* at distances under 10 μm, perhaps reflecting the presence of microbe-free patches in the images leading to a modest apparent clustering relative to a random distribution. Cross-correlations at distances below 0.5 microns were negative, reflecting the inability of different cells to occupy the same physical space. We did not carry out spatial arrangement analysis for cells of *Bacteroidetes* spp. because these cells consisted largely of filamentous forms that likely would have extended from the biofilm into the surrounding seawater in life but were flattened across the biofilm in the whole-mount preparations (e.g., Fig. 3A–C), so their spatial arrangement in images would not accurately reflect their arrangement in vivo.

**Table 3:** Spatial correlation of major taxa on tip biofilm using linear dipole analysis in *daime*

Probe	Distance of maximum spatial autocorrelation (μm)	Distance of maximum spatial cross-correlation (μm)
*Granulosicoccus*	1.5	NA
*Verrucomicrobia/Planctomycetes*	1.1	NA
*Alphaproteobacteria*	1.1	NA
*Granulosicoccus* vs. *Verrucomicrobia/Planctomycetes*	NA	4.7 to 7.4
*Granulosicoccus* vs. *Alphaproteobacteria*	NA	4.7
*Alphaproteobacteria* vs. *Verrucomicrobia/Planctomycetes*	NA	1.1
*Bacteroidetes*	NA	NA

### Unidentified bacteria attach to *Granulosicoccus* cells

**Fig. 9:**
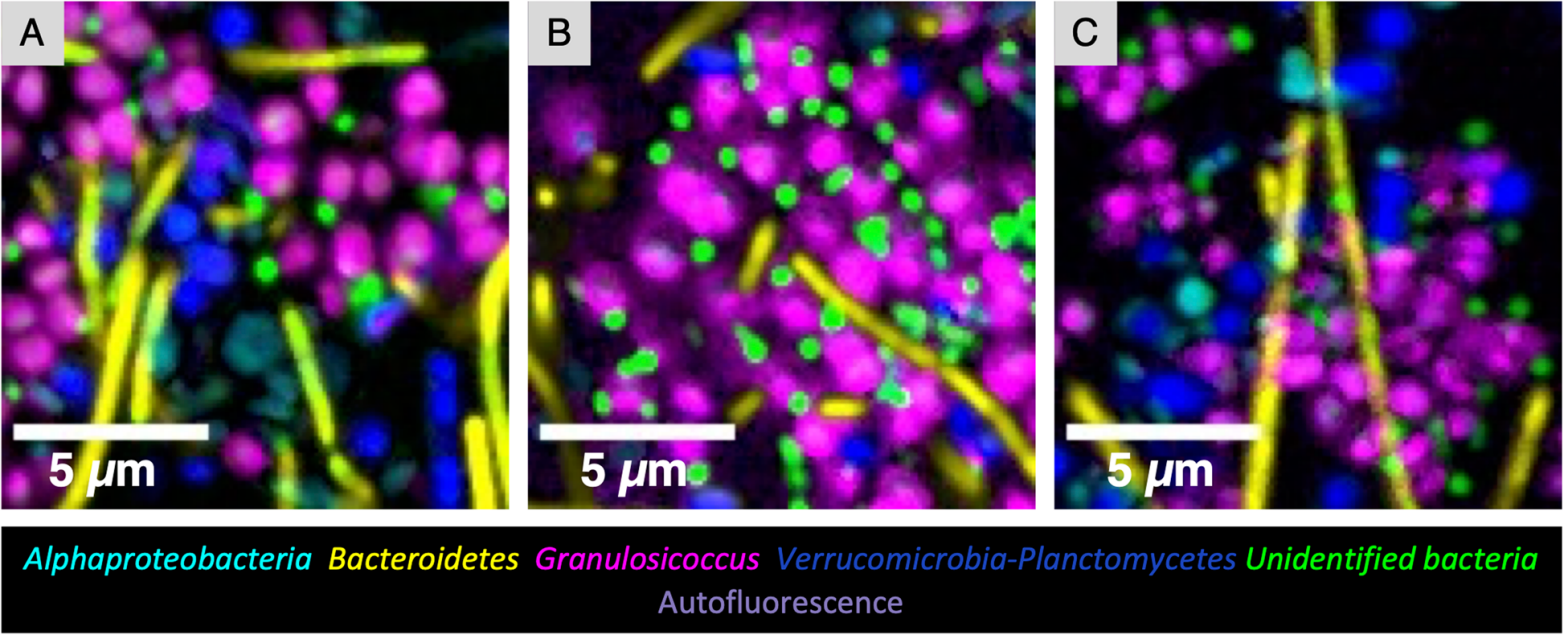
Epibiotic bacteria associated with *Granulosicoccus* cells. Small bacteria identified only with the near-universal Eub338-1 probe were observed adjacent to *Granulosicoccu*s despite being surrounded by numerous bacteria of different taxa. **A**-**C** Representative images of samples collected on different dates during the summer on Tatoosh Island

In some images, we detected bacteria that were identified only with the near-universal but not any of the taxon-specific probes, and which showed a presumably epibiotic lifestyle. They stood out for their small size and their position consistently adjacent to *Granulosicoccus* cells (Fig. 9A–C). These small bacteria were observed in samples collected on different dates during the summer on Tatoosh Island. Interestingly, despite being surrounded by numerous cells of different taxa, they were always observed touching *Granulosicoccus*, suggesting a specific attachment to this taxon. Further research is necessary to determine whether the relationship between these taxa is symbiotic or parasitic.

## Discussion

The micron-scale spatial organization we observed provides evidence about the nature of the interactions between disparate members of this complex microbiota. Imaging results show that the different microbes do not colonize the host in separate, distinct patches, as might be expected if colonization and growth by one microbe produced strong local inhibition of colonization by other microbes or if the host presented spatially distinct patches of binding sites or niches for colonization of different microbes. Instead, microbial distribution was nearly random at micrometer scales, with a modest but significant tendency for *Verrucomicrobia*/*Planctomycetes* and *Alphaproteobacteria* to be more closely associated than random at distances between 1 and 5 μm. Thus, the spatial organization of the microbiota is consistent with syntrophy or metabolic interaction between disparate microbes.

Our results suggest that competition for space is not a primary driver of community structure in this system. Colonization is sparse in samples from young tissue near the basal meristem, with apparently ample host surface available for microbial attachment. Even on the older tissue at the tip, we observed a patchy biofilm in which direct access to the host tissue appeared to be available. Thus, models such as the competitive lottery model [29], which require that the first colonizer can physically or metabolically exclude later colonizers, appear not to be supported. Instead, the micron-scale organization within the biofilm suggests that the different microbes may enhance rather than inhibit one another’s growth. There is increasing evidence that host-associated microbes have reciprocal, positive effects in marine ecosystems [30]. For example, a possible mechanism for such a growth-enhancing effect is the provision of different essential nutrients by different members of the community. A recent study shows that in a marine microbial community *Rhodobacterales* (*Alphaproteobacteria*) synthesize vitamin B-12, *Flavobacteria* (*Bacteroidetes*) synthesize vitamin B-7, and *Verrucomicrobia* synthesize vitamin B-1 [31]. These same groups are represented in the kelp microbiota imaged here, and such metabolic interdependencies may underlie the community structure we observe.

### Functional implications of the kelp microbiota

The functional importance of the dense microbial biofilm revealed through CLASI-FISH is relatively unknown, but the position of the biofilm at the interface between the host tissue and the surrounding water column suggests the possibility that it modulates light and nutrient availability to the host kelp, influences biofouling, and participates in host-microbe metabolic exchange. At this interface, mucilage production by kelps may play a critical role in providing structure for surface-associated microbes. By associating with macroalgal surfaces, microbes likely benefit from a predictable source of dissolved organic matter [5] and a persistent substrate for colonization. In turn, potential benefits of these microbes to the host kelp may include generation of antibacterial compounds that protect the host against biofouling and pathogens [1, 11, 32–34] or competitors [35], or the metabolisms that provision nutrients and vitamins to the host [4, 9].

Imaging revealed close association, at micrometer scales, of different microbial taxa with one another and with the host, a spatial organization that creates the conditions necessary for metabolic exchange among microbes [36] and between host and microbiota. While recent studies have described microbial communities in association with kelp through genomics [11, 37–39], the metabolic role of the microbes relative to the host has yet to be clarified. Possible functional interactions between macroalgae and their epibionts, both detrimental and potentially beneficial, have been the subject of several recent reviews [40–43]. Nutrient exchanges between host and microbes are functionally significant in phytoplankton (e.g., [44]). *N. luetkeana* at Tatoosh Island release ~ 16% of fixed carbon into the surrounding seawater as dissolved organic carbon (DOC) [5], a quantity consistent with DOC release estimates for other kelp species [45, 46]. By living on the kelp surface, biofilm microbes are presented with a consistent and labile metabolic resource, in addition to the structural kelp polymers that kelp microbes can degrade [47]. The release of carbohydrate exudates likely favors heterotrophic microbial metabolisms, and the *Granulosicoccus* sp. sequence variant in this study shares 97% sequence identity to *Granulosicoccus antarcticus* [48], which is a heterotrophic microbe that contains urease and both nitrate and nitrite reductase genes [49]. As a heterotroph, *Granulosicoccus* sp. likely uses the abundant DOC, while nitrogen transformation genes suggest nitrogen metabolisms, including ammonification, that may impact the host kelp. The finding that *Granulosicoccus* is ubiquitous across the Atlantic Ocean on the foundational brown alga species *Fucus vesiculosus* [50] suggests it could be a core taxon for marine macrophytes. Likewise, studies of microbial nutrient transformation in near-shore waters of Tatoosh Island showed that these microbial nitrogen metabolisms were strongest in association with the surfaces of a red alga, *Prionitis sternbergii*, rather than in seawater or associated with inert substrates [51]. This finding suggests that epibiont communities on algae are enriched for microbes carrying out ammonium oxidation and nitrate reduction, both of which might serve to retain and recycle dissolved inorganic nitrogen near the surface of the alga. Future work combining analysis of spatial organization through CLASI-FISH with microbial metagenomics or functional assays will provide insight into the functional importance of the kelp microbiome.

### Comparison of the *Nereocystis* microbiota to other microbiomes

CLASI-FISH revealed the microbiota of *N. luetkeana* to be dense and complex. This result is consistent with previous work by investigators who used standard FISH to highlight individual taxa within the bacterial community growing on or within macroalgal fronds [52–54]. The simultaneous identification of the major groups enabled by CLASI-FISH permits analysis of the relative distribution of the major groups and their potential for direct spatial interaction at micron scales. Our results lay out a framework that can be furthered by future experiments using more complex probe sets.

The microbial biofilm visualized here is notable for its high density but moderate thickness. In the most densely colonized samples, our images show a biofilm several cells thick and comparable to the density of 10^7^–10^8^ cells/cm^2^ measured for the brown alga *Fucus vesiculosus* [55] and 10^3^–10^7^ cells/cm^2^ for *Laminaria hyperborea* [56]. Other marine organisms have a lower surface colonization density; for example, the density of microbes in coral mucus is estimated at only 10^5^–10^6^ per cm^3^ [57, 58], on the same order of magnitude as typical microbial density in seawater. The thickness of the kelp microbiota, in the range of 10 microns, is small compared to the potentially hundreds of microns in thickness reached by biofilms in other settings characterized by fluid flow and regular abrasion, such as the human mouth [59–61].

### Kelp biofilm thickness and its relationship to biofilm dynamics and host health

The limited thickness of the microbial biofilm on *N. luetkeana* raises questions about mechanisms by which the thickness of the biofilm may be limited. We hypothesize that there may be a dynamic process of biofilm loss and re-growth over weeks to months, resulting in overall stability in biofilm structure and thickness at the tip of the blade throughout the growing season. This loss may occur via host shedding of the mucilaginous coating to which the biofilm is adhered. Alternatively, biofilm thickness may be limited by the intrinsic rate of growth of the microbes, by grazing of the biofilm by micro- or macro-invertebrates [62], or by lateral spread of the underlying kelp blade surface itself. Future studies with highly resolved time course sampling or live imaging will be necessary to gain a comprehensive understanding of the dynamic processes shaping the kelp microbiome; nonetheless, comparing the micron-scale spatial structure of new and old kelp tissues provided some insight into the process of microbiome community assembly. Rather than a well-mixed community that might result if assembly occurred through the continual settlement of microbes onto kelp tissue, we found a spatially structured biofilm with compositional distinctions between microbes associated with the kelp surface and microbes at the outer edge of the biofilm. We also observed clustering of *Granulosicoccus* sp. cells that are likely the result of growth and cell division. Finally, a phylogenetic analysis of our previous 16S sequencing results revealed that the *N. luetkeana* microbiome from both new and older kelp tissues is comprised of taxa that are phylogenetically clustered relative to the regional species pool [10]; thus, community assembly is non-random and the kelp surface may be acting as a selective filter for the specific taxonomic groups that we visualized here. Further investigations into the specific functions of these microbes will reveal their overall importance to the kelp microbiome.

Shifts in microbial composition between healthy and stressed macroalgae have been reported [9], but the low density of bacteria on the kelp from Squaxin Island was unexpected, as we had initially assumed that a population in decline would be more likely to be overrun with microbes than nearly devoid of them. *N. luetkeana* from Squaxin Island released DOC at a lower rate compared to those from Tatoosh Island [5]; however, further research is necessary to determine the relationship between DOC exudation and microbial cell density. The lack of microbes on individuals from the declining Squaxin population may reflect a host that is not provisioning the ideal resources for bacterial growth. Alternatively, it may result from stress-related sloughing of the mucus layer, or it may reflect the influence of the environment on the microbiota at each location. The extent to which potentially stressed macroalgae are associated with a distinct and depauperate microbiome certainly deserves further study, especially as kelp forests continue to decline in South Puget Sound [21] and in many locations globally [63].

The majority of the bacterial epibionts at Squaxin were shown by both sequencing and imaging to be *Alphaproteobacteria*, represented by a single highly-abundant amplicon sequence variant from the genus *Robiginitomaculum*, family *Hyphomonadaceae*. *Alphaproteobacteria* from the order *Rhizobiales* produce unipolar adhesins which are essential for cell-cell adhesion, biofilm formation, and effective root colonization [64, 65]. The exopolysaccharide N-acetylglucosamine, synthesized by bacterial cells, plays an important role in biofilm formation in *Staphylococcus aureus* [66, 67] and *Escherichia coli* [68]. The consistent presence of N-acetylglucosamine or N-acetylmuramic acid residues at one end of *Alphaproteobacteria* cells suggests that it may be involved in cell adhesion to the kelp surface, and it may be indicative of the prostheca structures of *Robiginitomaculum* sp. The high relative abundance of *Alphaproteobacteria* (*Robiginitomaculum* sp*.*) in Squaxin kelp samples, in which the observed bacterial density is low, might reflect their potential to attach to the kelp surface more permanently or more readily than other members of the microbiota.

## Conclusions

Our detailed imaging with CLASI-FISH has shown that photosynthetic blades of the canopy-forming kelp *Nereocystis luetkeana* host a complex microbial biofilm that is both dense and spatially differentiated. Within this community, microbes of different taxa are in intimate cell-to-cell contact with one another; microbial cells invade the interior of kelp cells as well as cover their external surfaces, and a subset of the surface microbiota projects into the water column. Close spatial associations highlight the potential for metabolic interactions between key members of the kelp microbiome as well as between microbes and their host. The biofilm coating the surface of the kelp blade is well-positioned to mediate interactions between the host and surrounding organisms and to modulate the chemistry of the surrounding water column. A mechanistic understanding of this symbiosis will broaden our understanding of host-microbiome interactions and potentially enable better management of macroalgae for conservation, restoration, aquaculture, biofuels, and biological carbon sequestration.

## Methods

### Sample collection and 16S rRNA gene sequencing

CLASI-FISH microscopy was carried out on a subset of samples analyzed previously with 16S rRNA gene sequencing and reported in Weigel and Pfister [10]. We carried out CLASI-FISH and imaging on a total of 15 samples: 12 from a persistent population at Tatoosh Island and 3 from a declining population at Squaxin Island (Additional file 2: Table S1). On Tatoosh Island, Washington in the United States (48° 23′ 37.0″ N 124° 44′ 06.5″ W), photosynthetic blade tissues of *N. luetkeana* were sampled at five time points spaced roughly 2 weeks apart (11 June–22 August 2017), spanning the peak in annual biomass. At each time point, two tissue samples (2 × 1 cm^2^) were collected from a single blade—one at the basal meristem, roughly 2 cm from where the blade connects to the stipe, to capture recently produced tissue (~ 24 to 48 h old) and another near the apical end of the blade tip to sample older tissue (weeks to months old). Samples were collected from different kelp individuals at each date. The 12 imaged samples from Tatoosh Island include samples from 8 kelp individuals: 4 pairs (*n* = 8 samples) from both the base (near the meristematic tissue) and the tip (older tissue) of the same kelp frond over the time series, and an additional 4 samples from the tip of different kelp blades in June and July (Additional file 2: Table S1). Total blade length and linear blade growth rates were measured to approximate the age of tissues sampled [10]. Kelp blade tissue samples were also collected from Squaxin Island, in the Southern Puget Sound (47° 10′ 38.7″ N 122° 54′ 42.2″ W) on 21 June 2017. At this site, kelp tissue samples were collected from the middle of the kelp blade, and samples from 3 individuals were selected for imaging. Kelp blade tissues for CLASI-FISH and 16S rRNA gene sequencing were collected together from adjacent locations on the kelp blade. Samples collected for CLASI-FISH were preserved in 95% ethanol and stored at – 20 °C, while samples for 16S rRNA gene sequencing had no preservatives and were temporarily frozen at – 20 °C until they were shipped to – 80°C. DNA for 16S rRNA gene sequencing was extracted from whole kelp tissues; details of DNA extraction, 16S rRNA gene sequencing, and sequence analysis are contained in Weigel and Pfister [10]. Sequence quality control and processing included chimera detection and removal, sequence error elimination, singleton exclusion, sequence trimming, removal of chloroplast and mitochondrial reads, and generation of amplicon sequence variants (ASVs) [10]. The relative abundance barplot (Fig. 1) was generated with unrarefied 16S sequence data due to low bacterial read counts in samples from the base of the kelp blade; bacterial read counts per sample after sequence quality control are listed in Additional file 2: Table S1.

### CLASI-FISH probe set design and validation

To investigate the spatial organization of the kelp microbiota we developed a probe set for CLASI-FISH (combinatorial labeling and spectral imaging–fluorescence in situ hybridization) including 7 oligonucleotide probes to enable simultaneous identification and imaging of the major bacterial groups. For comprehensive coverage of bacteria, we used the probes Eub338-I, II, and III [22, 23] using one fluorophore for probe Eub338-I and a different fluorophore to label both Eub338-II and Eub338-III, to differentiate the *Verrucomicrobia* and *Planctomycetes* from the rest of the Bacteria. For greater taxonomic resolution within the bacteria identified by Eub338-I, the probe set contained probes specific for the major groups comprising the *N. luetkeana* microbiota: *Bacteroidetes*, *Alphaproteobacteria*, and *Gammaproteobacteria*. For added taxonomic resolution within *Gammaproteobacteria*, we designed two new probes targeting the most abundant genus, *Granulosicoccus*. Thus, the probe set targeted nested levels of taxonomic identification, with cells identified by combinations of one, two, or three fluorophores. Probes used in this study are listed in Table 1.

Probes for the genus *Granulosicoccus* were designed based on prior amplicon sequencing results. An alignment of the most abundant amplicon sequence variants (ASVs) from all taxa in the community, plus all the *Granulosicoccus* ASVs, was performed using Geneious 11.1.3. The alignment was reviewed manually to look for candidate regions for probe design. We selected candidate probes which were a perfect match to most or all *Granulosicoccus* sequences and which had at least two strong mismatches to all other taxa. Predicted hybridization efficiency on target and non-target taxa was then evaluated using mathFISH [69], a tool for thermodynamic modeling of hybridization efficiency of RNA-targeted probes, and probe target position and length were adjusted to maximize the predicted specificity of hybridization. Based on these results, two probes were designed which we used interchangeably: Gran670 (5′-CACCGCTACACCCGGAATTCCGC-3′) and Gran737 (5′-TCAGCGTCAGTATTGTTCCAGA-3′). We tested probe specificity by applying the set of 7 probes (Eub338-I, Eub338-II, Eub338-III, Alf968, Gam42a, Bac1058, and Gran670 or Gran737) to 5 pure cultures and imaged the cultures under the same conditions as kelp samples. Each probe hybridized with its target taxa (positive control) and only faint hybridization was observed with nontarget taxa (negative control, Additional file 7: Fig. S6).

### Bacterial strains and growth conditions

*Granulosicoccus coccoides* DSM 25245 and *G. antarcticus* DSM 24912 were cultured in Bacto Marine Broth media (DIFCO 2216) (pH 7.5 and 7 for *G. coccoides* and *G. antarcticus*, respectively). Cultures were incubated with agitation (180 rpm) at 25 °C or 21 °C for 2 (*G. coccoides*) or 7 days (*G. antarcticus*). Cells were fixed with 2% PFA on ice for 90 min, washed, transferred to 50% ethanol, and stored at – 20 °C until use.

### Embedding and sectioning for imaging

For methacrylate embedding, kelp samples stored in 95% ethanol at – 20 °C were placed in 100% ethanol for 30 min followed by acetone for 1 hour, infiltrated with Technovit 8100 glycol methacrylate (EMSdiasium.com) infiltration solution for 3 h (replacing with fresh solution every hour), followed by a final infiltration overnight at 4^o^C. Samples were then transferred to Technovit 8100 embedding solution and solidified for 12 h at 4 °C. Blocks were sectioned to 5 μm thickness using a Leica microtome (RM2145) and sections were applied to Ultrastick slides (Thermo Scientific). Sections were stored at room temperature.

### Combinatorial labeling and spectral imaging fluorescence in situ hybridization

We used two methods to visualize the spatial structure of the kelp microbiota: whole-mount agarose preparations and methacrylate sections. When possible, we used pieces of the same kelp sample for both methods. For the whole-mount-agarose method, one piece of kelp was placed on a slide and 50 μl of 1% low-melting-point agarose was dropped on it and the sample was allowed to cool on ice for 10 min before the CLASI-FISH procedure. Methacrylate sections were subject to CLASI-FISH directly on slides.

Hybridization solution [900 mM NaCl, 20 mM Tris, pH 7.5, 0.01% SDS, 20% (vol/vol) formamide, each probe at a final concentration of 2 μM] was applied to kelp pieces and incubated at 46 °C for 2 h in a chamber humidified with 20% (vol/vol) formamide. Whole-mount-agarose preparations were maintained in horizontal position and were washed with 100 μl of pre-warmed wash buffer (215 mM NaCl, 20 mM Tris, pH 7.5, 5mM EDTA) five times at RT followed by three washes with 500 μl of wash buffer at 48 °C for 5 min each. The hybridization conditions were the same for methacrylate sections, but washing was carried out by incubating the slides in 50 ml of washing buffer for 15 minutes at 48 °C. Samples were then incubated with wheat germ agglutinin (20 μg ml^−1^) conjugated with Alexa Fluor 680 at room temperature for 30 min in the dark. Agarose-coated samples were washed with 100 μl of sterile cold water three times. Excess agarose was cut off with a disinfected razor. Methacrylate sections were washed by dipping the slide into 50 ml of ice-cold water to remove excess salt. Samples were mounted in ProLong Gold antifade reagent (Invitrogen) with a #1.5 coverslip and cured overnight in the dark at room temperature before imaging.

### Image acquisition and linear unmixing

Spectral images were acquired using a Carl Zeiss LSM 780 confocal microscope with a Plan-Apochromat 40×, 1.4 N.A. oil immersion objective. Images were captured using simultaneous excitation with 405-, 488-, 561-, and 633-nm laser lines. Linear unmixing was performed using ZEN Black software (Carl Zeiss) using reference spectra acquired from cultured cells hybridized with Eub338-I probe labeled with one of the 6 fluorophores in the probe set and imaged as above. Unmixed images were assembled and false-colored using FIJI software [70].

### Cell quantification and spatial analysis of CLASI-FISH images

Cell quantification was carried out using FIJI software [70]. A 3 × 3 median filter was applied to the images to reduce noise and preserve edges. The images were then thresholded using the Bernsen method for the *Alphaproteobacteria* channel and the IsoData method for the *Granulosicoccus*, *Bacteroidetes*, and *Verrucomicrobia*/*Planctomycetes* channels. In parallel, a binary mask was generated from the original 16-bit image using the “Find maxima” function with “segmented particles” output type. The binary mask was pasted onto the thresholded image to separate adjacent cells. Occasionally, we observed inconsistent hybridization signal resulting from cross-reaction of probes with non-target cells; for example, *Alphaproteobacteria* cells hybridized with the Eub338-I probe as expected, but we sometimes observed a reaction of the *Alphaproteobacteria* probe with cells that hybridized not with Eub338-I but with the Eub338-II and III probes for *Verrucomicrobia* and *Planctomycetes* (Additional file 8: Fig. S7). To exclude cells with such signals from quantification, we removed them from the segmented images for the relevant channel. As an additional check, for each segmented cell (or ROI, region of interest) we assessed its intensity in each probe channel. We set a threshold to discriminate between ambiguous and conclusive signal: when the mean signal intensity of the ROI in the channel of the expected taxon was at least 2× its intensity in any of the other channels, it was considered conclusively identified. Cell abundance, size, and density measurements were generated using only conclusively identified cells.

We used the software *daime* [28] to analyze the spatial structure of the biofilm from the tip of *N. luetkeana* blades (*n* = 8 kelp individuals, *n* = 20 images total). We imported the segmented images for each probe channel separately as “tif” files. Image size was 212.55 μm × 212.55 μm and 2048 × 2048 pixels. For stereological analysis, we quantified spatial distribution using the 2D linear dipole algorithm for analysis of single and multiple populations. Software settings were to use “scan whole reference space” to a distance of 50 μm, record the correlation at every 0.15 μm, and to classify very small objects and overlapping regions between different taxa as noise. *Bacteroidetes* were excluded from the *daime* analysis because most biomass of this taxon in our images consisted of filaments that became flattened onto the sample during the FISH and mounting procedure, which is not their original location in the biofilm and would result in inaccurate conclusions.

## Supplementary Information

**Additional file 1: Figure S1.** Probes for 4 major groups collectively identify most bacterial cells on kelp samples. (A, D and G) show the signal from 4 group-specific probes (*Alphaproteobacteria*, *Bacteroidetes*, *Granulosicoccus* and *Verrucomicrobia + Planctomycetes*) each labeled with a different fluorophore. (B, E, and H): Signal from Eub338-I (green) and Eub338-II + Eub338-III (blue) collectively identifying most bacteria. (C, F and I): Overlay shows that most bacteria hybridizing with Eub338 are also identified by one of the 4 group-specific probes; only a small number of cells are labeled only with the Eub338-I probe and are otherwise unidentified (red ovals). Collection date is shown at left. Probe names and target taxon names (in parentheses) are shown at bottom of each column.

**Additional file 2: Table S1.** Samples of *N. luetkeana* kelp used for CLASI-FISH, their collection date and location, total blade length, estimated tissue age, total number of 16S rRNA gene sequences from each sample, number of 16S rRNA gene sequences identified as bacterial, and the percent of all 16S rRNA gene sequences that were identified as bacterial rather than host chloroplast. Note that samples from the base of the blade had a low proportion of bacterial sequences compared to those from the top of the blade.

**Additional file 3: Figure S2.** Strategy for sample preparation and orientation. (A) We used both whole-mounts and embedding and sectioning as complementary preparation methods on portions of the same samples to confirm and validate findings on spatial organization of the kelp biofilm. Pieces of kelp frond, over the course of hybridization and washing steps, sometimes came apart into their component layers. We found that coating the tissue with a layer of agarose helped the sample to remain intact during manipulations. In addition to whole-mount preparations, we employed an alternative preparation procedure in which the fixed sample was embedded in methacrylate resin followed by sectioning and FISH. This procedure minimized distortion and preserved spatial organization by immobilizing the sample in the resin. It permitted imaging of thin cross-sections through the kelp blade, with the benefit of providing a clear view of the biofilm on both surfaces and interior of the blade. (B) In many whole-mount images the sample is tilted relative to the plane of imaging, such that the confocal microscope image is an optical section through the sample at an oblique angle and a single plane of focus captures both the kelp blade and the overlying microbial community (Fig. 2C). Illustration not to scale.

**Additional file 4: Figure S3.** Bacterial abundance on surface of old and young tissue in *N. luetkeana.* (A) Cell abundance differed depending on collection site and portion of the kelp blade sampled. Cell counts per field of view (FOV) are shown as boxplots showing all data points as well as the median (line) and first and third quartiles for each taxon; n = 20 FOV for Tatoosh base and tip and 16 FOV for Squaxin. Bacterial abundance on older tissue (tip) is higher compared to young tissue (base) or declining kelp (Squaxin). Collection site and portion of the kelp blade sampled (Tip; Base; Mid = middle) are indicated in the x-axis labels. (B) Relative abundance estimated by imaging is comparable to that estimated by 16S rRNA gene sequencing. Relative abundance based on total cell counts from individual samples is shown; n = 2 to 9 FOV per sample. Sample numbers (cf. Table S1) and portion of the kelp blade sampled (T = tip, B = Base, M = middle) are indicated in the x-axis labels. Collection site is shown at the bottom. Abundance estimates from imaging and from 16S rRNA gene sequencing (Fig. 1) were similar despite the different methods and different scale of sampling: imaging was from fields of view of 0.04 mm^2^ while DNA extraction for sequencing was from an entire tissue sample of 2 cm^2^ [10].

**Additional file 5: Figure S4.** Bacterial abundance close to and far from kelp surface. Taxon abundance changes depending on distance from the kelp surface. All taxa were more abundant near the kelp and diminished far from the surface; *Granulosicoccus* made up a larger fraction of the community near the surface than near the water column. Cell counts were measured from z-stack images of whole mounts where the sample was flat enough that most cells in the field of view were a similar distance from the kelp surface, and where the *z*-stack was thick enough to clearly distinguish cells close to and farther from the kelp surface. Two fields of view from different samples collected on the same day (July 10) satisfied these criteria. From these *z*-stacks we selected the plane containing microbes closest to the kelp surface and the plane 5 to 6 micrometers farther toward the water column, which was the last plane in which microbes were abundant. Cells were segmented and identified to taxon and the abundance of each taxon at each distance (mean and range) is shown.

**Additional file 6: Figure S5.** Linear dipole analysis quantifies clustering of major taxa in whole-mount images of the kelp surface*.* The correlation function (dark line) and 95% confidence intervals (shaded regions) are shown. The pair correlation values indicate to what degree the taxa are positively or negatively correlated at each distance. Values >1 indicate attraction, <1 indicate repulsion and =1 indicate random distribution. (A) Each taxon showed significant autocorrelation indicating a tendency of cells to form single-taxon clusters. *Granulosicoccus* cells showed maximum autocorrelation at 1.5 μm with a typical cluster size (estimated by the full width at half maximum) of 1 to 3 μm. *Verrucomicrobia* and *Alphaproteobacteria* cells each showed maximum autocorrelation at approximately 1 μm. (B) Spatial cross-correlation between different taxa was modest. Peak cross-correlation between *Alphaproteobacteria* and *Verrucomicrobia* occurred at 1 μm. The slightly elevated cross-correlation between *Granulosicoccus* and *Alphaproteobacteria* and between *Granulosicoccus* and *Verrucomicrobia* at distances under 10 μm may reflect the presence of microbe-free patches in the images, leading to a modest apparent clustering relative to a random distribution of cells throughout the image. The mean (solid line) and 95% confidence interval (ribbon) are shown based on 8 individual kelp with 2 to 3 censuses (fields of view) from each.

**Additional file 7: Figure S6.** Validation matrix of a set of 7 probes. Each probe was labeled with a distinct fluorophore except for Eub338-II and Eub338-III which were both labeled with the fluorophore Dy415. To validate the probes for specificity, we applied the set of probes to pure cultures, hybridized and imaged under the same conditions as kelp samples. Results show that each specific probe hybridized with its expected target taxa; some cross-reactions are visible (e.g., Gam42a probe with *Bacteroidetes* cells) but are faint relative to hybridization of those same cells with the probe targeting them (e.g., Bac1058 probe with *Bacteroidetes* cells). Probe name is shown at top of each column. Bacterial culture names are shown in left column. Target taxon for each probe is shown in row in the bottom.

**Additional file 8: Figure S7.** Faint cross-reaction of *Alphaproteobacteria* probe. (A, B and C) show hybridization of a kelp sample with Eub338-I almost-universal, Eub338-II/III *Verrucomicrobia*-*Planctomycetes* (hereafter referred to as simply *Verrucomicrobia*) and Alf968 *Alphaproteobacteria* probes, respectively. (D): Merged image of panels (A) and (B) showing non-overlapping signals demonstrates that the Eub338-I and Eub338-II/III probes hybridize with different and mutually exclusive target cells. (E) Merged image of panels (B) and (C) showing that some cells hybridize with both Eub338-II/III and Alf968 probes. (F) Merged image of panels (A), (B) and (C) showing that some cells hybridize with both *Alphaproteobacteria* and Eub338-I probes as expected, while others hybridize with both *Alphaproteobacteria* and *Verrucomicrobia* probes suggesting that there is a slight nonspecific reaction of the *Alphaproteobacteria* probe with *Verrucomicrobia*. Probe names are shown below each panel. Target taxon name is shown in parentheses.

**Additional file 9: Table S2.** Each ASV is shown together with its representative sequence, taxonomic identification, and distribution across sequenced samples. This is a subset of the 16S rRNA sequence data from Weigel and Pfister 2019 [10].

## Data Availability

Raw 16S rRNA gene sequences are available at the National Center for Biotechnology Information’s Sequence Read Archive and the European Bioinformatics Institute (accession # PRJEB29319 on both servers). A list of samples used in this study, including sequence repository sample IDs, is contained in Additional file 2: Table S1. The imaging data generated and analyzed during the current study are available from the corresponding author on reasonable request.

## References

[1] Egan S, Harder T, Burke C, Steinberg P, Kjelleberg S, Thomas T (2013). The seaweed holobiont: understanding seaweed-bacteria interactions. FEMS Microbiol Rev.

[2] McFall-Ngai M, Hadfield MG, Bosch TC (2013). Animals in a bacterial world, a new imperative for the life sciences. Proc Natl Acad Sci U S A.

[3] Berg G, Grube M, Schloter M, Smalla K (2014). Unraveling the plant microbiome: looking back and future perspectives. Front Microbiol.

[4] Croft MT, Lawrence AD, Raux-Deery E, Warren MJ, Smith AG (2005). Algae acquire vitamin B12 through a symbiotic relationship with bacteria. Nature.

[5] Weigel BL, Pfister CA (2021). The dynamics and stoichiometry of dissolved organic carbon release by kelp. Ecology.

[6] Martin M, Barbeyron T, Martin R, Portetelle D, Michel G, Vandenbol M (2015). The cultivable surface microbiota of the brown alga *Ascophyllum nodosum* is enriched in macroalgal-polysaccharide-degrading bacteria. Front Microbiol.

[7] Lin JD, Lemay MA, Parfrey LW (2018). Diverse bacteria utilize alginate within the microbiome of the Giant Kelp *Macrocystis pyrifera*. Front Microbiol.

[8] Thomas F, Le Duff N, Wu TD (2021). Isotopic tracing reveals single-cell assimilation of a macroalgal polysaccharide by a few marine Flavobacteria and Gammaproteobacteria. ISME J.

[9] Tarquinio F, Bourgoure J, Koenders A, Laverock B, Säwström C, Hyndes GA (2018). Microorganisms facilitate uptake of dissolved organic nitrogen by seagrass leaves. ISME J.

[10] Weigel BL, Pfister CA (2019). Successional dynamics and seascape-level patterns of microbial communities on the canopy-forming kelps *Nereocystis luetkeana* and *Macrocystis pyrifera*. Front Microbiol.

[11] Michelou VK, Caporaso JG, Knight R, Palumbi SR (2013). The Ecology of microbial communities associated with *Macrocystis pyrifera*. PLoS One.

[12] Chen M, Parfrey L (2018). Incubation with macroalgae induces large shifts in water column microbiota, but minor changes to the epibiota of co-occurring macroalgae. Mol Ecol.

[13] Hollants J, Leliaert F, De Clerck O, Willems A (2013). What we can learn from sushi: a review on seaweed-bacterial associations. FEMS Microbiol Ecol.

[14] Kolenbrander PE, Palmer RJ, Periasamy S, Jakubovics NS (2010). Oral multispecies biofilm development and the key role of cell–cell distance. Nat Rev Microbiol.

[15] Cordero OX, Datta MS (2016). Microbial interactions and community assembly at microscales. Curr Opin Microbiol.

[16] Dal Co A, van Vliet S, Kiviet DJ, Schlegel S, Ackermann M (2020). Short-range interactions govern the dynamics and functions of microbial communities. Nat Ecol Evol.

[17] Zhang Z, van Kleunen M, Becks L, Thakur MP (2020). Towards a general understanding of bacterial interactions. Trends Microbiol.

[18] Valm AM, Mark Welch JL, Rieken CW, Hasegawa Y, Sogin ML, Oldenbourg R, Dewhirst FE, Borisy GG (2011). Systems-level analysis of microbial community organization through combinatorial labeling and spectral imaging. Proc Natl Acad Sci U S A.

[19] Valm AM, Mark Welch JL, Borisy GG (2012). CLASI-FISH: principles of combinatorial labeling and spectral imaging. Syst Appl Microbiol.

[20] Pfister CA, Berry HD, Mumford T (2018). The dynamics of kelp forests in the Northeast Pacific Ocean and the relationship with environmental drivers. J Ecol.

[21] Berry HD, Mumford TF, Christiaen B (2021). Long-term changes in kelp forests in an inner basin of the Salish Sea. PLoS One.

[22] Amann RI, Binder BJ, Olson RJ, Chisholm SW, Devereux R, Stahl DA (1990). Combination of 16S rRNA-targeted oligonucleotide probes with flow cytometry for analyzing mixed microbial populations. Appl Environ Microbiol.

[23] Daims H, Brühl A, Amann R, Schleifer KH, Wagner M (1999). The domain-specific probe EUB338 is insufficient for the detection of all bacteria: development and evaluation of a more comprehensive probe set. Syst Appl Microbiol.

[24] Neef A (1997). Anwendung der in situ-Einzelzell- Identifizierung von Bakterien zur Populations analyse in komplexen mikrobiellen Biozönosen [Doctoral thesis].

[25] Manz W, Amann R, Ludwig W, Wagner M, Schleifer KH (1992). Phylogenetic oligodeoxynucleotide probes for the major subclasses of *Proteobacteria*: problems and solutions. Syst Appl Microbiol.

[26] Schlundt C, Mark Welch JL, Knochel AM, Zettler ER, Amaral-Zettler LA (2020). Spatial structure in the “Plastisphere”: molecular resources for imaging microscopic communities on plastic marine debris. Mol Ecol Resour.

[27] Lee K, Lee HK, Choi TH, Cho JC (2007). *Robiginitomaculum antarcticum* gen. nov., sp. nov., a member of the family *Hyphomonadaceae*, from Antarctic seawater. Int J Syst Evol Microbiol.

[28] Daims H, Lücker S, Wagner M (2006). *daime*, a novel image analysis program for microbial ecology and biofilm research. Environ Microbiol.

[29] Burke C, Steinberg P, Rusch D, Kjelleberg S, Thomas T (2011). Bacterial community assembly based on functional genes rather than species. Proc Natl Acad Sci U S A.

[30] Wilkins LGE, Leray M, O’Dea A (2019). Host-associated microbiomes drive structure and function of marine ecosystems. PLoS Biol.

[31] Gómez-Consarnau L, Sachdeva R, Gifford SM (2018). Mosaic patterns of B-vitamin synthesis and utilization in a natural marine microbial community. Environ Microbiol.

[32] Rao D, Webb JS, Holmström C, Case R, Low A, Steinberg P, Kjelleberg S (2007). Low densities of epiphytic bacteria from the marine alga *Ulva australis* inhibit settlement of fouling organisms. Appl Environ Microbiol.

[33] Tebben J, Motti C, Tapiolas D, Thomas-Hall P, Harder T (2014). A coralline algal-associated bacterium, *Pseudoalteromonas* strain J010, yields five new korormicins and a bromopyrrole. Mar Drugs.

[34] Lee STM, Davy SK, Tang SL, Kench PS (2016). Mucus sugar content shapes the bacterial community structure in thermally stressed *Acropora muricata*. Front Microbiol.

[35] Barott KL, Rohwer FL (2012). Unseen players shape benthic competition on coral reefs. Trends Microbiol.

[36] Seth EC, Taga ME (2014). Nutrient cross-feeding in the microbial world. Front Microbiol.

[37] Minich JJ, Morris MM, Brown M, Doane M, Edwards MS, Michael TP, Dinsdale EA (2018). Elevated temperature drives kelp microbiome dysbiosis, while elevated carbon dioxide induces water microbiome disruption. PLoS One.

[38] Lemay MA, Martone PT, Keeling PJ, Burt JM, Krumhansl KA, Sanders RD, Wegener Parfrey L (2018). Sympatric kelp species share a large portion of their surface bacterial communities: Kelp-associated bacterial diversity. Environ Microbiol.

[39] Qiu Z, Coleman MA, Provost E, Campbell AH, Kelaher BP, Dalton SJ, Thomas T, Steinberg PD, Marzinelli EM (2019). Future climate change is predicted to affect the microbiome and condition of habitat-forming kelp. Proc Royal Soc B Biol Sci.

[40] Wahl M, Goecke F, Labes A, Dobretsov S, Weinberger F (2012). The second skin: ecological role of epibiotic biofilms on marine organisms. Front Microbiol.

[41] Ramanan R, Kim BH, Cho DH, Oh HM, Kim HS (2016). Algae-bacteria interactions: evolution, ecology and emerging applications. Biotechnol Adv.

[42] Singh RP, Reddy CR (2016). Unraveling the functions of the macroalgal microbiome. Front Microbiol.

[43] Florez JZ, Camus C, Hengst MB, Buschmann AH (2017). A functional perspective analysis of macroalgae and epiphytic bacterial community interaction. Front Microbiol.

[44] Amin SA, Hmelo LR, van Tol HM, Durham BP, Carlson LT, Heal KR, Morales RL, Berthiaume CT, Parker MS, Djunaedi B, Ingalls AE, Parsek MR, Moran MA, Armbrust EV (2015). Interaction and signalling between a cosmopolitan phytoplankton and associated bacteria. Nature.

[45] Abdullah MI, Fredriksen S (2004). Production, respiration and exudation of dissolved organic matter by the kelp *Laminaria hyperborea* along the west coast of Norway. J Mar Biol Assoc UK.

[46] Reed DC, Carlson CA, Halewood ER, Nelson JC, Harrer SL, Rassweiler A, Miller RJ (2015). Patterns and controls of reef-scale production of dissolved organic carbon by giant kelp *Macrocystis pyrifera*: DOC production by giant kelp. Limnol Oceanogr.

[47] Bengtsson MM, Sjøtun K, Storesund JE, Øvreas L (2011). Utilization of kelp-derived carbon sources by kelp surface-associated bacteria. Aquat Microb Ecol.

[48] Lee K, Lee HK, Choi TH, Kim KM, Cho JC (2007). *Granulosicoccaceae* fam. nov, to include *Granulosicoccus antarcticus* gen. nov, sp. nov, a non-phototrophic, obligately aerobic chemoheterotroph in the order *Chromatiales*, isolated from Antarctic seawater. J Microbiol Biotechnol.

[49] Kang I, Lim Y, Cho JC (2018). Complete genome sequence of *Granulosicoccus antarcticus* type strain IMCC3135^T^, a marine gammaproteobacterium with a putative dimethylsulfoniopropionate demethylase gene. Mar Genomics.

[50] Capistrant-Fossa KA, Morrison HG, Engelen AH, et al. The microbiome of the habitat-forming brown alga *Fucus vesiculosus* (Phaeophyceae) has similar cross-Atlantic structure that reflects past and present drivers. J Phycol. 2021. 10.1111/jpy.13194. 10.1111/jpy.13194 34176151

[51] Pfister CA, Altabet MA (2019). Enhanced microbial nitrogen transformations in association with macrobiota from the rocky intertidal. Biogeosciences.

[52] Tujula NA, Holmström C, Mussmann M, Amann R, Kjelleberg S, Crocetti GR (2006). A CARD-FISH protocol for the identification and enumeration of epiphytic bacteria on marine algae. J Microbiol Methods.

[53] Tujula NA, Crocetti GR, Burke C, Thomas T, Holmström C, Kjelleberg S (2010). Variability and abundance of the epiphytic bacterial community associated with a green marine Ulvacean alga. ISME J.

[54] Bengtsson MM, Øvreås L (2010). *Planctomycetes* dominate biofilms on surfaces of the kelp *Laminaria hyperborea*. BMC Microbiol.

[55] Stratil SB, Neulinger SC, Knecht H, Friedrich AK, Wahl M (2013). Temperature-driven shifts in the epibiotic bacterial community composition of the brown macroalga *Fucus vesiculosus*. Microbiologyopen.

[56] Bengtsson MM, Sjøtun K, Øvreås L (2010). Seasonal dynamics of bacterial biofilms on the kelp *Laminaria hyperborea*. Aquat Microb Ecol.

[57] Garren M, Azam F (2010). New method for counting bacteria associated with coral mucus. Appl Environ Microbiol.

[58] Glasl B, Herndl GJ, Frade PR (2016). The microbiome of coral surface mucus has a key role in mediating holobiont health and survival upon disturbance. ISME J.

[59] Zijnge V, van Leeuwen MB, Degener JE, Abbas F, Thurnheer T, Gmür R, Harmsen HJ (2010). Oral biofilm architecture on natural teeth. PLoS One.

[60] Mark Welch JL, Rossetti BJ, Rieken CW, Dewhirst FE, Borisy GG (2016). Biogeography of a human oral microbiome at the micron scale. Proc Natl Acad Sci U S A.

[61] Wilbert SA, Mark Welch JL, Borisy GG (2020). Spatial ecology of the human tongue dorsum microbiome. Cell Rep.

[62] Chenelot H, Konar B (2007). *Lacuna vincta* (Mollusca, Neotaenioglossa) herbivory on juvenile and adult *Nereocystis luetkeana* (Heterokontophyta, Laminariales). Hydrobiologia.

[63] Filbee-Dexter K, Wernberg T (2018). Rise of turfs: a new battlefront for globally declining kelp forests. BioScience.

[64] Fritts RK, LaSarre B, Stoner AM, Posto AL, McKinlay JB (2017). A *Rhizobiales*-specific unipolar polysaccharide adhesin contributes to *Rhodopseudomonas palustris* biofilm formation across diverse photoheterotrophic conditions. Appl Environ Microbiol.

[65] Williams A, Wilkinson A, Krehenbrink M, Russo DM, Zorreguieta A, Downie JA (2008). Glucomannan-mediated attachment of *Rhizobium leguminosarum* to pea root hairs is required for competitive nodule infection. J Bacteriol.

[66] Izano EA, Amarante MA, Kher WB, Kaplan JB (2008). Differential roles of poly-N-acetylglucosamine surface polysaccharide and extracellular DNA in *Staphylococcus aureus* and *Staphylococcus epidermidis* biofilms. Appl Environ Microbiol.

[67] Lin MH, Shu JC, Lin LP, Chong KY, Cheng YW, Du JF, Liu ST (2015). Elucidating the crucial role of poly N-acetylglucosamine from *Staphylococcus aureus* in cellular adhesion and pathogenesis. PLoS One.

[68] Wang X, Preston JF, Romeo T (2004). The pgaABCD locus of *Escherichia coli* promotes the synthesis of a polysaccharide adhesin required for biofilm formation. J Bacteriol.

[69] Yilmaz LS, Parnerkar S, Noguera DR (2011). *mathfish*, a web tool that uses thermodynamics-based mathematical models for in *silico* evaluation of oligonucleotide probes for fluorescence *in situ* hybridization. Appl Environ Microbiol.

[70] Schindelin J, Arganda-Carreras I, Frise E, Kaynig V, Longair M, Pietzsch T, Preibisch S, Rueden C, Saalfeld S, Schmid B, Tinevez JY, White DJ, Hartenstein V, Eliceiri K, Tomancak P, Cardona A (2012). Fiji: an open-source platform for biological-image analysis. Nat Methods.

