# Supplementary material for: Spatial organization of the kelp microbiome at micron scales
Source: Microbiome. 2022 Mar 24;10:52. doi: 10.1186/s40168-022-01235-w (PMC8944128; doi:10.1186/s40168-022-01235-w)
Supplement: Supplementary file 2 — Additional file 1: Figure S1. Probes for 4 major groups collectively identify most bacterial cells on kelp samples. (A, D and G) show the signal from 4 group-specific probes (Alphaproteobacteria, Bacteroidetes, Granulosicoccus and Verrucomicrobia + Planctomycetes) each labeled with a different fluorophore. (B, E, and H): Signal from Eub338-I (green) and Eub338-II + Eub338-III (blue) collectively identifying most bacteria. (C, F and I): Overlay shows that most bacteria hybridizing with Eub338 are also identified by one of the 4 group-specific probes; only a small number of cells are labeled only with the Eub338-I probe and are otherwise unidentified (red ovals). Collection date is shown at left. Probe names and target taxon names (in parentheses) are shown at bottom of each column. [file 40168_2022_1235_MOESM2_ESM.pdf]

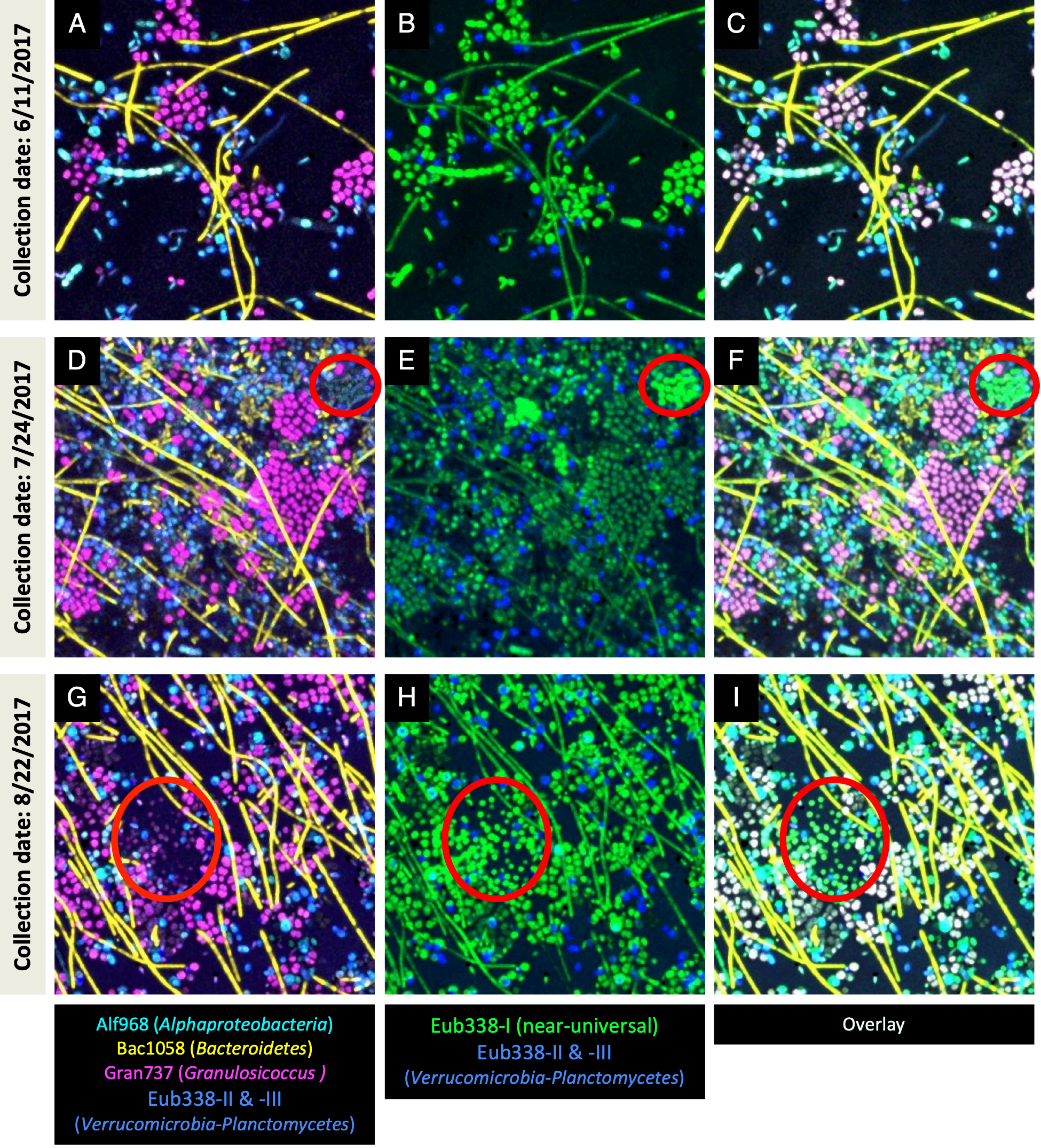
Collection date: 6/11/2017
Collection date: 7/24/2017
Collection date: 8/22/2017
A
B
C
D
E
F
G
H
I
Alf968 (*Alphaproteobacteria*)
Bac1058 (*Bacteroidetes*)
Gran737 (*Granulosicoccus*)
Eub338-II & -III
(*Verrucomicrobia-Planctomycetes*)
Eub338-I (near-universal)
Eub338-II & -III
(*Verrucomicrobia-Planctomycetes*)
Overlay
