# Supplementary material for: Spatial organization of the kelp microbiome at micron scales
Source: Microbiome. 2022 Mar 24;10:52. doi: 10.1186/s40168-022-01235-w (PMC8944128; doi:10.1186/s40168-022-01235-w)
Supplement: Supplementary file 3 — Additional file 2: Table S1. Samples of N. luetkeana kelp used for CLASI-FISH, their collection date and location, total blade length, estimated tissue age, total number of 16S rRNA gene sequences from each sample, number of 16S rRNA gene sequences identified as bacterial, and the percent of all 16S rRNA gene sequences that were identified as bacterial rather than host chloroplast. Note that samples from the base of the blade had a low proportion of bacterial sequences compared to those from the top of the blade. [file 40168_2022_1235_MOESM3_ESM.docx]

**Table S1**.

| Sample ID | Sequence repository Sample ID | Blade sample type | Date collected | Location collected | Total blade length (cm) | Estimated age tissue (days) | Total Sequence Count | Bacterial Sequence Count | % Bacterial 16S Seqs |
| --- | --- | --- | --- | --- | --- | --- | --- | --- | --- |
| 63-B | 12016.63B | base | 6/11/17 | Tatoosh | 124 | < 7 | 27,927 | 429 | 1.5 |
| 63-A | 12016.63A | tip | 6/11/17 | Tatoosh | 124 | 62 | 42,113 | 34,443 | 81.8 |
| 61-B | 12016.61A | tip | 6/11/17 | Tatoosh | 95 | 48 | 51,654 | 46,278 | 89.6 |
| 81-B | 12016.81B | base | 6/25/17 | Tatoosh | 87 | < 7 | 32,046 | 1,190 | 3.7 |
| 81-A | 12016.81A | tip | 6/25/17 | Tatoosh | 87 | 44 | 51,006 | 45,742 | 89.7 |
| 82-A | 12016.82A | tip | 6/25/17 | Tatoosh | 131 | 66 | 50,981 | 45,680 | 89.6 |
| 128-A | 12016.128A | tip | 7/10/17 | Tatoosh | 102 | 51 | 48,165 | 42,433 | 88.1 |
| 132-A | 12016.132A | tip | 7/10/17 | Tatoosh | 156 | 78 | 73,121 | 65,537 | 89.6 |
| 158-B | 12016.158B | base | 7/24/17 | Tatoosh | 146 | < 7 | 31,388 | 729 | 2.3 |
| 158-A | 12016.158A | tip | 7/24/17 | Tatoosh | 146 | 73 | 61,338 | 59,489 | 97.0 |
| 271-B | 12016.271B | base | 8/22/17 | Tatoosh | 72 | < 7 | 14,014 | 213 | 1.5 |
| 271-A | 12016.271A | tip | 8/22/17 | Tatoosh | 72 | 36 | 34,566 | 3,635 | 10.5 |
| 71 | 12016.71 | mid-blade | 6/21/17 | Squaxin | 268 | 67 | 41,277 | 14,142 | 34.3 |
| 72 | 12016.72 | mid-blade | 6/21/17 | Squaxin | 266 | 67 | 39,558 | 3,011 | 7.6 |
| 73 | 12016.73 | mid-blade | 6/21/17 | Squaxin | 302 | 76 | 42,700 | 4,302 | 10.1 |
