# Supplementary figures and images for: Spatial organization of the kelp microbiome at micron scales
Source: Microbiome. 2022 Mar 24;10:52. doi: 10.1186/s40168-022-01235-w (PMC8944128; doi:10.1186/s40168-022-01235-w)

## A Sample preparation

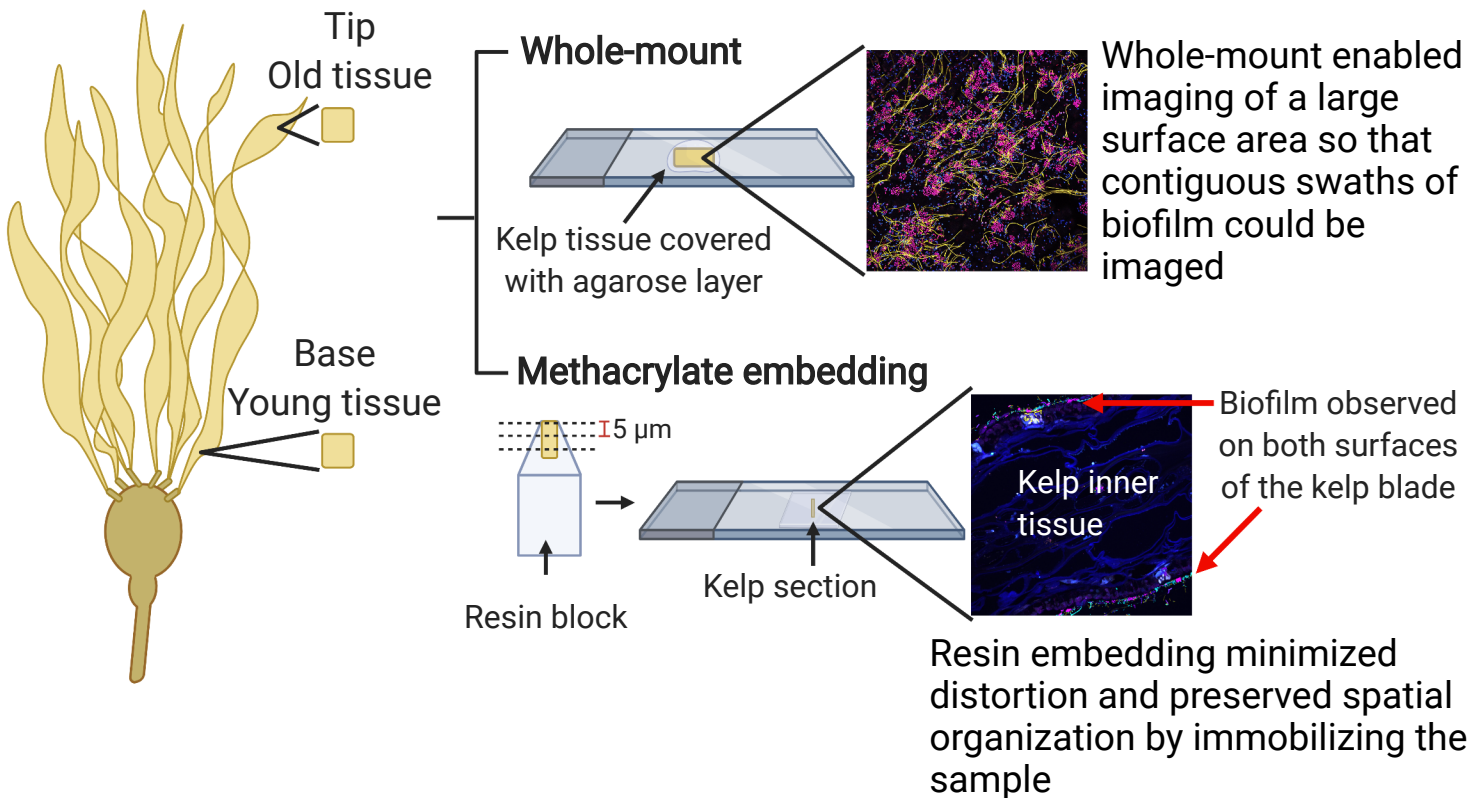

## B Oblique viewing angle in tilted sample

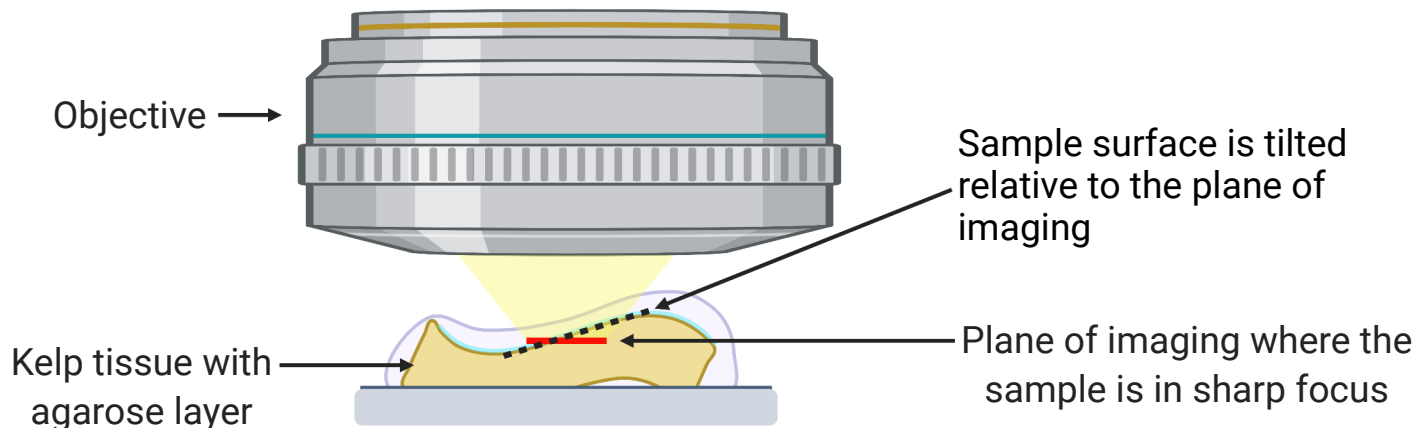

Supplement: Supplementary file 4 — Additional file 3: Figure S2. Strategy for sample preparation and orientation. (A) We used both whole-mounts and embedding and sectioning as complementary preparation methods on portions of the same samples to confirm and validate findings on spatial organization of the kelp biofilm. Pieces of kelp frond, over the course of hybridization and washing steps, sometimes came apart into their component layers. We found that coating the tissue with a layer of agarose helped the sample to remain intact during manipulations. In addition to whole-mount preparations, we employed an alternative preparation procedure in which the fixed sample was embedded in methacrylate resin followed by sectioning and FISH. This procedure minimized distortion and preserved spatial organization by immobilizing the sample in the resin. It permitted imaging of thin cross-sections through the kelp blade, with the benefit of providing a clear view of the biofilm on both surfaces and interior of the blade. (B) In many whole-mount images the sample is tilted relative to the plane of imaging, such that the confocal microscope image is an optical section through the sample at an oblique angle and a single plane of focus captures both the kelp blade and the overlying microbial community (Fig. 2C). Illustration not to scale. [file 40168_2022_1235_MOESM4_ESM.pdf]

**A**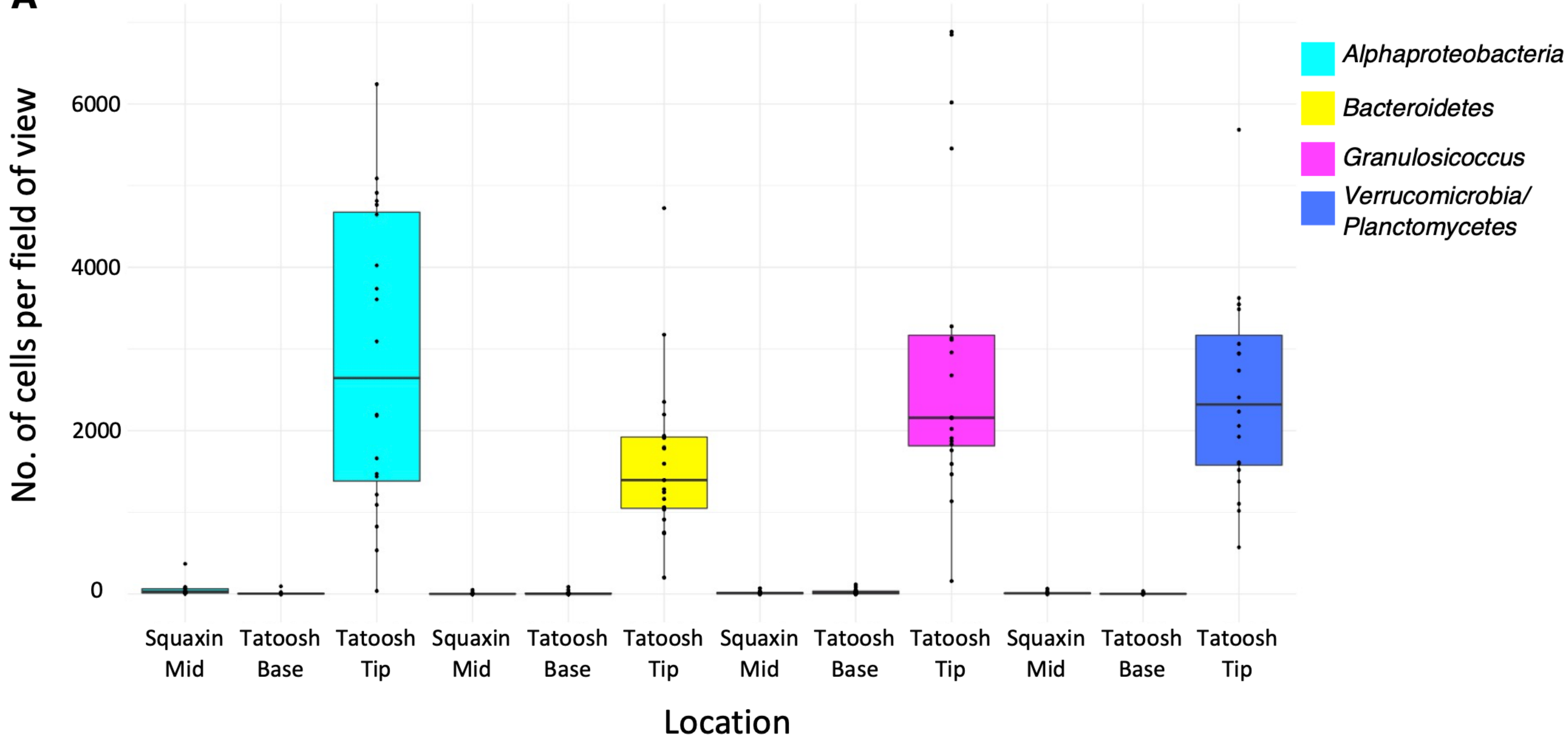**B**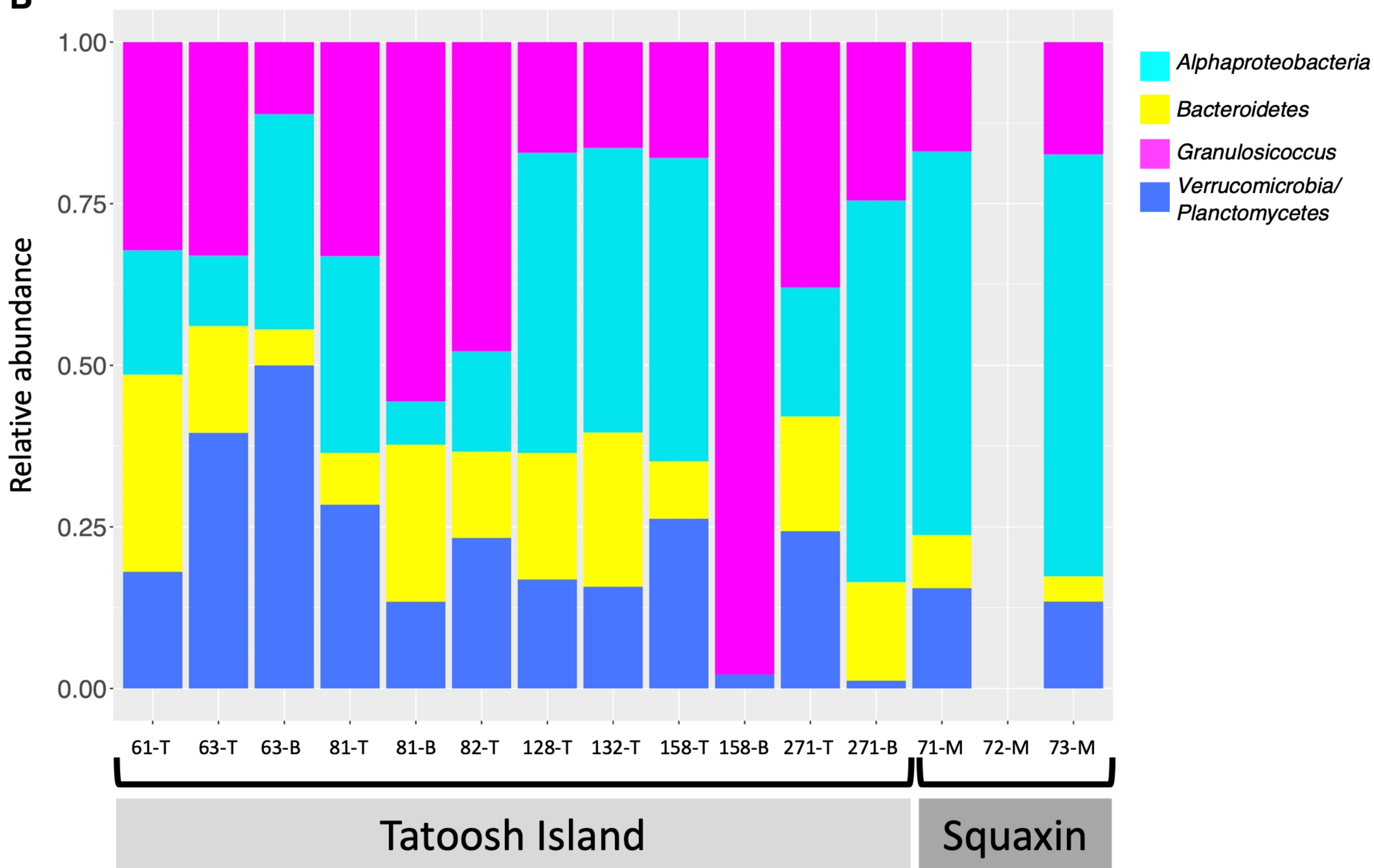

Supplement: Supplementary file 5 — Additional file 4: Figure S3. Bacterial abundance on surface of old and young tissue in N. luetkeana. (A) Cell abundance differed depending on collection site and portion of the kelp blade sampled. Cell counts per field of view (FOV) are shown as boxplots showing all data points as well as the median (line) and first and third quartiles for each taxon; n = 20 FOV for Tatoosh base and tip and 16 FOV for Squaxin. Bacterial abundance on older tissue (tip) is higher compared to young tissue (base) or declining kelp (Squaxin). Collection site and portion of the kelp blade sampled (Tip; Base; Mid = middle) are indicated in the x-axis labels. (B) Relative abundance estimated by imaging is comparable to that estimated by 16S rRNA gene sequencing. Relative abundance based on total cell counts from individual samples is shown; n = 2 to 9 FOV per sample. Sample numbers (cf. Table S1) and portion of the kelp blade sampled (T = tip, B = Base, M = middle) are indicated in the x-axis labels. Collection site is shown at the bottom. Abundance estimates from imaging and from 16S rRNA gene sequencing (Fig. 1) were similar despite the different methods and different scale of sampling: imaging was from fields of view of 0.04 mm2 while DNA extraction for sequencing was from an entire tissue sample of 2 cm2 [10]. [file 40168_2022_1235_MOESM5_ESM.pdf]

A

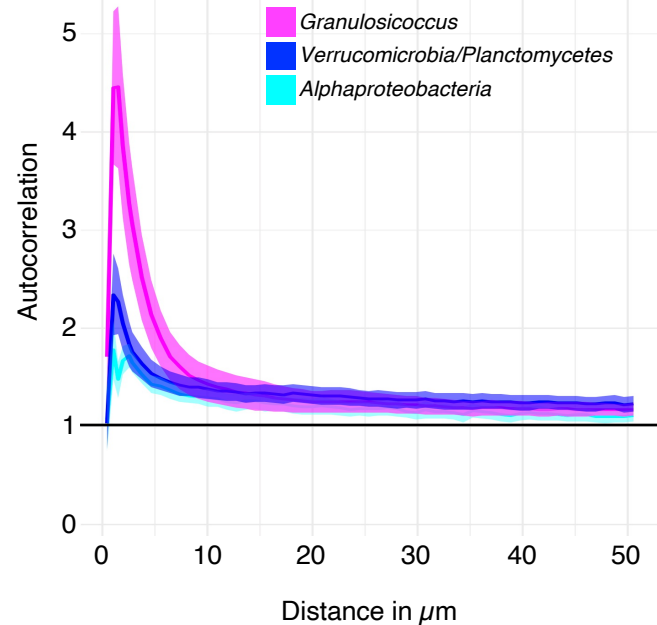

B

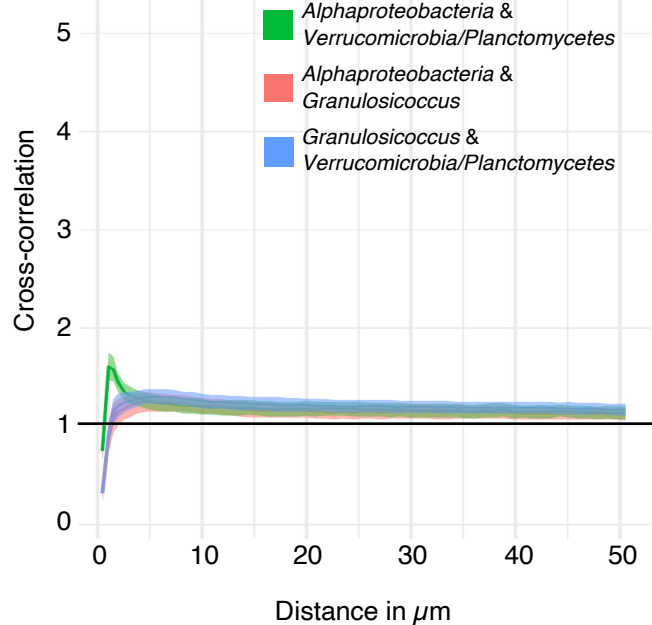

Supplement: Supplementary file 7 — Additional file 6: Figure S5. Linear dipole analysis quantifies clustering of major taxa in whole-mount images of the kelp surface. The correlation function (dark line) and 95% confidence intervals (shaded regions) are shown. The pair correlation values indicate to what degree the taxa are positively or negatively correlated at each distance. Values >1 indicate attraction, <1 indicate repulsion and =1 indicate random distribution. (A) Each taxon showed significant autocorrelation indicating a tendency of cells to form single-taxon clusters. Granulosicoccus cells showed maximum autocorrelation at 1.5 μm with a typical cluster size (estimated by the full width at half maximum) of 1 to 3 μm. Verrucomicrobia and Alphaproteobacteria cells each showed maximum autocorrelation at approximately 1 μm. (B) Spatial cross-correlation between different taxa was modest. Peak cross-correlation between Alphaproteobacteria and Verrucomicrobia occurred at 1 μm. The slightly elevated cross-correlation between Granulosicoccus and Alphaproteobacteria and between Granulosicoccus and Verrucomicrobia at distances under 10 μm may reflect the presence of microbe-free patches in the images, leading to a modest apparent clustering relative to a random distribution of cells throughout the image. The mean (solid line) and 95% confidence interval (ribbon) are shown based on 8 individual kelp with 2 to 3 censuses (fields of view) from each. [file 40168_2022_1235_MOESM7_ESM.pdf]
