# Supplementary material for: Spatial organization of the kelp microbiome at micron scales
Source: Microbiome. 2022 Mar 24;10:52. doi: 10.1186/s40168-022-01235-w (PMC8944128; doi:10.1186/s40168-022-01235-w)
Supplement: Supplementary file 6 — Additional file 5: Figure S4. Bacterial abundance close to and far from kelp surface. Taxon abundance changes depending on distance from the kelp surface. All taxa were more abundant near the kelp and diminished far from the surface; Granulosicoccus made up a larger fraction of the community near the surface than near the water column. Cell counts were measured from z-stack images of whole mounts where the sample was flat enough that most cells in the field of view were a similar distance from the kelp surface, and where the z-stack was thick enough to clearly distinguish cells close to and farther from the kelp surface. Two fields of view from different samples collected on the same day (July 10) satisfied these criteria. From these z-stacks we selected the plane containing microbes closest to the kelp surface and the plane 5 to 6 micrometers farther toward the water column, which was the last plane in which microbes were abundant. Cells were segmented and identified to taxon and the abundance of each taxon at each distance (mean and range) is shown. [file 40168_2022_1235_MOESM6_ESM.pdf]

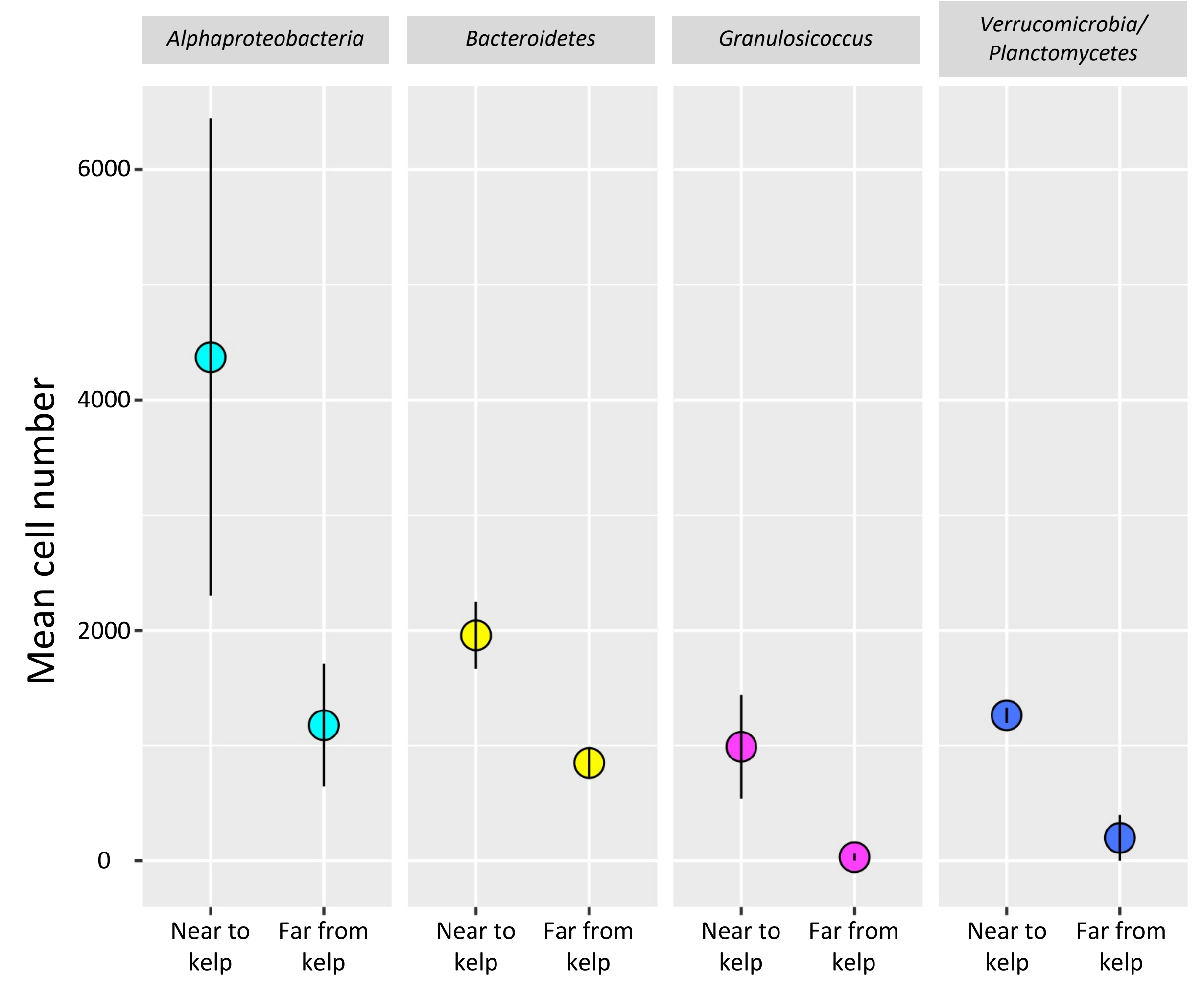

Alphaproteobacteria
Bacteroidetes
Granulosicoccus
Verrucomicrobia/ Planctomycetes
Mean cell number
6000
4000
2000
0
Near to kelp
Far from kelp
