# Supplementary material for: Spatial organization of the kelp microbiome at micron scales
Source: Microbiome. 2022 Mar 24;10:52. doi: 10.1186/s40168-022-01235-w (PMC8944128; doi:10.1186/s40168-022-01235-w)
Supplement: Supplementary file 8 — Additional file 7: Figure S6. Validation matrix of a set of 7 probes. Each probe was labeled with a distinct fluorophore except for Eub338-II and Eub338-III which were both labeled with the fluorophore Dy415. To validate the probes for specificity, we applied the set of probes to pure cultures, hybridized and imaged under the same conditions as kelp samples. Results show that each specific probe hybridized with its expected target taxa; some cross-reactions are visible (e.g., Gam42a probe with Bacteroidetes cells) but are faint relative to hybridization of those same cells with the probe targeting them (e.g., Bac1058 probe with Bacteroidetes cells). Probe name is shown at top of each column. Bacterial culture names are shown in left column. Target taxon for each probe is shown in row in the bottom. [file 40168_2022_1235_MOESM8_ESM.pdf]

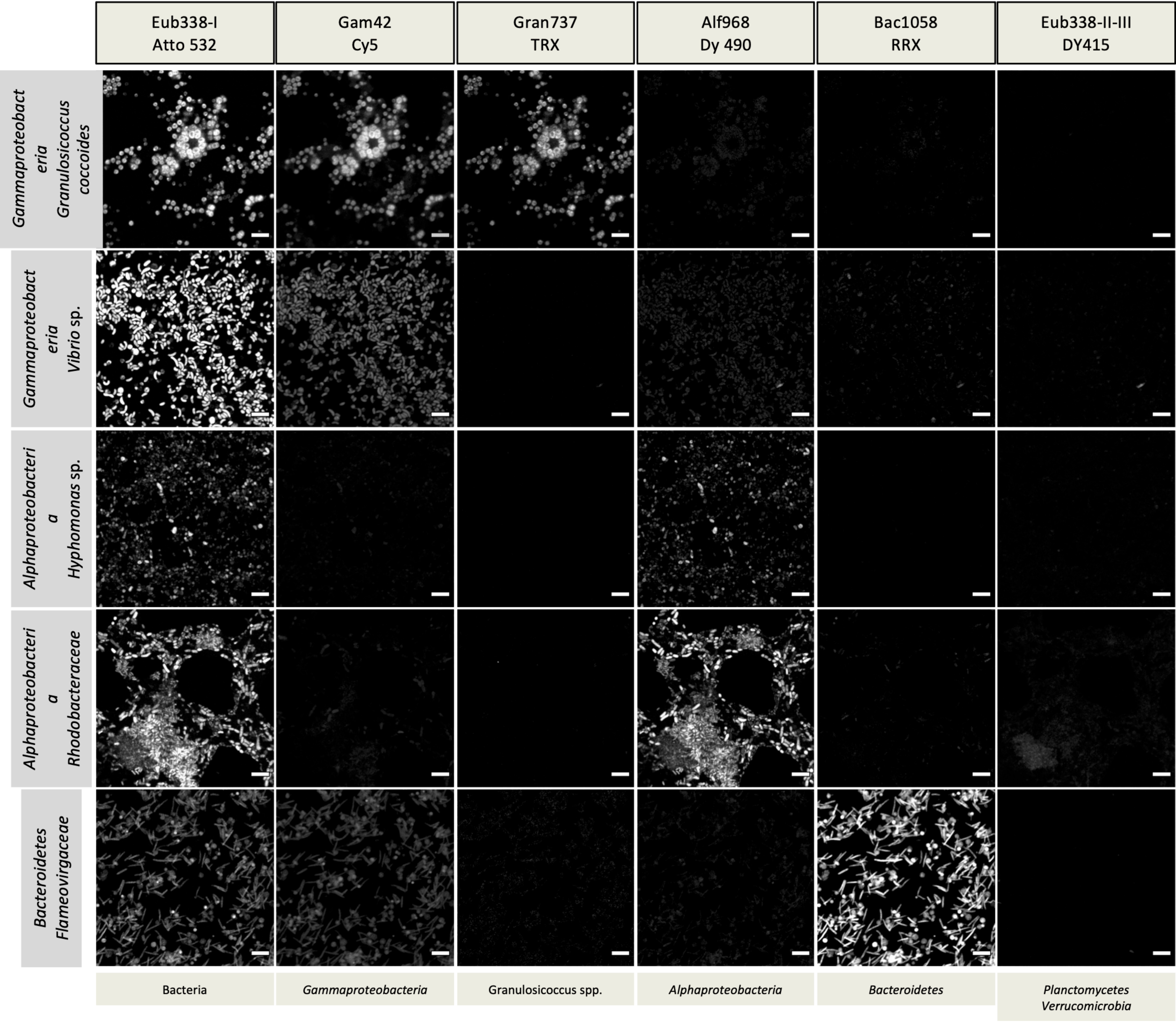

Eub338-I
Atto 532
Gam42
Cy5
Gran737
TRX
Alf968
Dy 490
Bac1058
RRX
Eub338-II-III
DY415
Gammaproteobacteria Granulosicoccus coccoides
Gammaproteobacteria Vibrio sp.
Alphaproteobacteria Hyphomonas sp.
Alphaproteobacteria Rhodobacteraceae
Bacteroidetes Flameovirgaceae
Bacteria
Gammaproteobacteria
Granulosicoccus spp.
Alphaproteobacteria
Bacteroidetes
Planctomycetes Verrucomicrobia
