# Supplementary material for: Spatial organization of the kelp microbiome at micron scales
Source: Microbiome. 2022 Mar 24;10:52. doi: 10.1186/s40168-022-01235-w (PMC8944128; doi:10.1186/s40168-022-01235-w)
Supplement: Supplementary file 9 — Additional file 8: Figure S7. Faint cross-reaction of Alphaproteobacteria probe. (A, B and C) show hybridization of a kelp sample with Eub338-I almost-universal, Eub338-II/III Verrucomicrobia-Planctomycetes (hereafter referred to as simply Verrucomicrobia) and Alf968 Alphaproteobacteria probes, respectively. (D): Merged image of panels (A) and (B) showing non-overlapping signals demonstrates that the Eub338-I and Eub338-II/III probes hybridize with different and mutually exclusive target cells. (E) Merged image of panels (B) and (C) showing that some cells hybridize with both Eub338-II/III and Alf968 probes. (F) Merged image of panels (A), (B) and (C) showing that some cells hybridize with both Alphaproteobacteria and Eub338-I probes as expected, while others hybridize with both Alphaproteobacteria and Verrucomicrobia probes suggesting that there is a slight nonspecific reaction of the Alphaproteobacteria probe with Verrucomicrobia. Probe names are shown below each panel. Target taxon name is shown in parentheses. [file 40168_2022_1235_MOESM9_ESM.pdf]

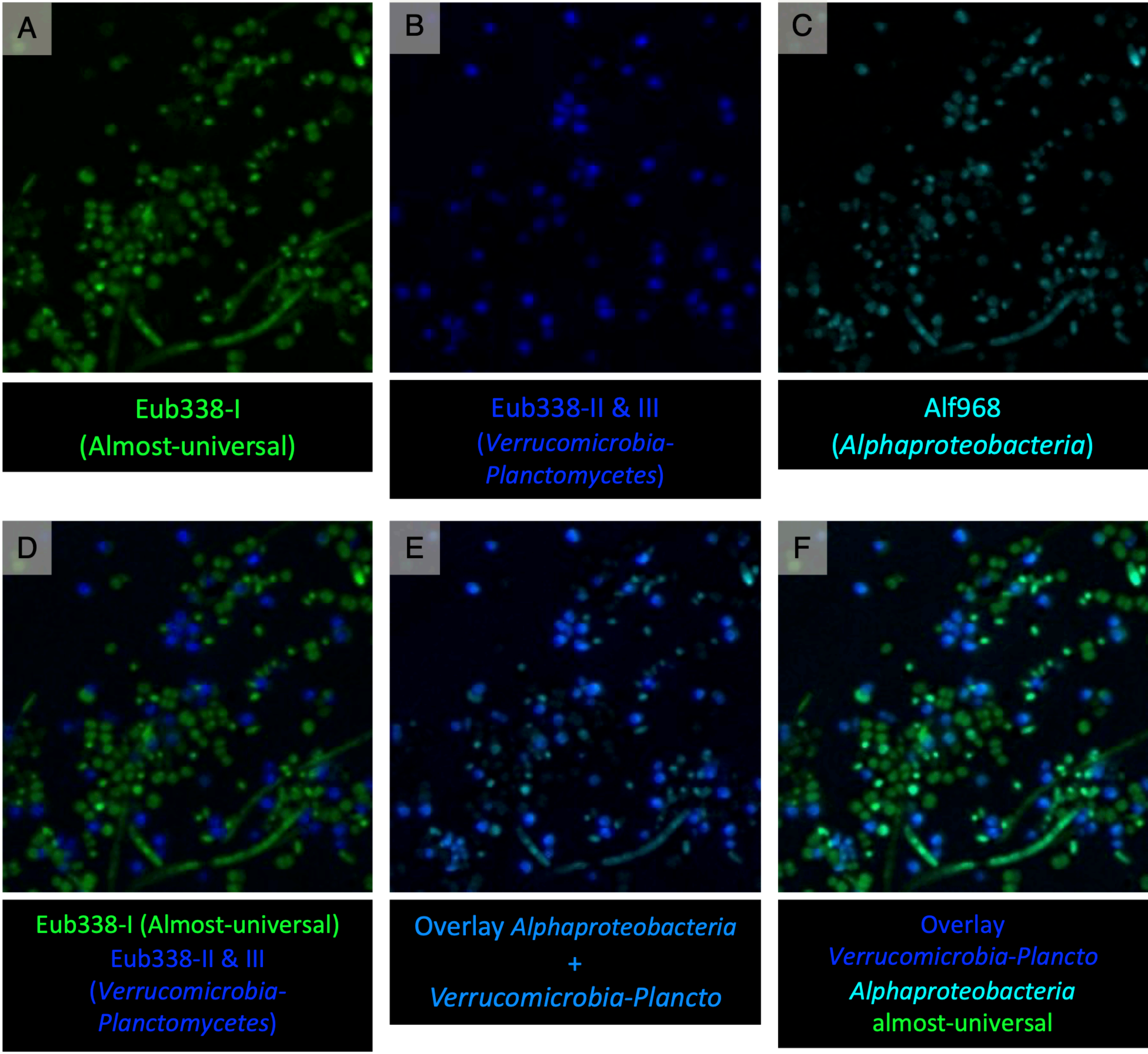
A
Eub338-I
(Almost-universal)
B
Eub338-II & III
(Verrucomicrobia-
Planctomycetes)
C
Alf968
(Alphaproteobacteria)
D
Eub338-I (Almost-universal)
Eub338-II & III
(Verrucomicrobia-
Planctomycetes)
E
Overlay Alphaproteobacteria
+
Verrucomicrobia-Plancto
F
Overlay
Verrucomicrobia-Plancto
Alphaproteobacteria
almost-universal
